# Phylogenetic, ecological and biomechanical constraints on larval form: A comparative morphological analysis of barnacle nauplii

**DOI:** 10.1371/journal.pone.0206973

**Published:** 2018-11-08

**Authors:** J. Y. Wong, K. Y. Karen Chan, Benny K. K. Chan

**Affiliations:** 1 Department of Life Science, National Taiwan Normal University, Taipei, Taiwan; 2 Biodiversity Program, Taiwan International Graduate Program, Academia Sinica, Taipei, Taiwan; 3 Biodiversity Research Center, Academia Sinica, Taipei, Taiwan; 4 Division of Life Science, The Hong Kong University of Science and Technology, Hong Kong SAR, China; 5 Biology Department, Swarthmore College, Swarthmore, PA, United States of America; Laboratoire de Biologie du Développement de Villefranche-sur-Mer, FRANCE

## Abstract

Barnacle naupliar larvae are differentiated from other zooplankton by their unique pair of frontal lateral horns, frontal filaments, and a pear-shaped cephalic shield. Their morphology impose constraints on their ecological functions and reflect their evolutionary history. To explore the potential functional basis underlying the similarities and differences in barnacle larval form, we conducted a meta-analysis on the shape of the barnacle nauplii’s cephalic shield and examined its relation to larval size, trophic mode, pelagic larval duration and habitat. Nauplii cephalic shield morphology of 102 species were quantified with normalized elliptic Fourier analysis. Most of the species were distributed around the center of the morphospace but a few extreme groups occupied the periphery: nauplii that were large and lecithotrophic. Subsequent principal component regression analyses showed that larval size was a good predictor of the first shape variations axis (aspect ratio). After allometry adjustment, nauplii from different trophic modes differentiated along the second axis of the major shape variations (relative frontal horn length). Habitat was a poor predictor of variations in naupliar body form, but it could be used to differentiate extreme morphology groups from other nauplii. Our result suggests that size-related biomechanical or developmental constraints and feeding requirements are important in shaping the evolution of the naupliar body form. Within the limitations of these functional constraints, habitat drives the divergence of extreme morphology groups from the majority of species. Our comparative morphometrics analysis demonstrated how variations in larval body form can be quantitatively linked to the functional needs that constrain or drive their diversity, and inform further empirical experiments on larval functional morphology.

## Introduction

The study of larval morphology has important phylogenetic and ecological implications. For example, similarities in larval characters between taxonomic groups have been used to support common ancestry at higher taxonomic levels, e.g. in crabs [1]. Nauplius larva was historically used to assign barnacles (Cirripedia) as crustacea; and larval characters, such as the presence of frontal horns, was used to support the monophyly of the barnacles [2]. Despite the essential role of barnacle larvae in systematics and population connectivity, little is known about the functional implications of the presence or absence of larval morphological variations between different species.

Functional constraints, the selective pressure exerted to perform various essential functions within the limits of development and biomechanics, are important forces that shape morphological evolution in marine invertebrate larvae [3]. Attempts have been made to connect overall larval morphology with ecological functions in barnacle larvae. For instance, the streamlined fusiform shape of cyprids was hypothesized to be an optimization for efficient swimming [4, 5] and the lack of streamlining in the shape of naupliar cephalic shield was hypothesized to help increase feeding efficiency [6].

While common functional needs could reduce the morphological variations between species, adaptation to specific habitats could drive larval morphology to diverge. For example, the exaggerated morphology of Lepadidae nauplii with very long frontal horns and tail processes is hypothesized to be an adaptation to the oligotrophic pelagic habitat [7]. However, unlike adult barnacles for which adaptive significance of morphological variations has been studied [8, 9, 10], little is known about if the same constraints and driving forces apply to their planktonic nauplii. A comprehensive, comparative study is needed to first provide a summary of morphological variations. Such data would in turn inform future experiments on functional morphology of barnacle larvae, putting the hypothetical relationships between larval form and functions to a test.

The inter-specific morphological variation in barnacle nauplius larvae has been reviewed [11, 12, 13]. The goal of these studies were often classification, and therefore they, only provide very detailed descriptions of morphological features of species from a single (or a few) target taxonomic group(s) and are often limited to a single locale, probably due to limited sample availability and/ or difficulty in preparing numerous detailed descriptions. Not only are comparisons rarely made between barnacle species from different families and habitats, variations in the overall larval body form—i.e. the shape of cephalic shield—have not been addressed. Landmark-based geometric morphometrics tools have been applied to study of marine invertebrate larvae [14] to quantitatively summarize larval shape changes. Such landmark-based tools typically require well-defined morphological characters to serve as landmarks. In the case of the cephalic shields of larval barnacles, where landmarks are hard to define, outline analysis can be applied [15].

We present a quantitative analysis on the shape diversity of cephalic shield of barnacle nauplius II larvae of 102 different species. Using outline analysis, we identified the major directions of shape change for the cephalic shield of nauplii and tested for differences in habitats or biomechanical requirements to perform the ecological functions of swimming and feeding, i.e. if size and trophic modes account for the observed variations.

## Materials and methods

### Data acquisition

Outlines of 102 species of barnacle nauplius larvae were analyzed. The majority of data were sourced from published descriptions (89 species from 63 publications) and the remaining 13 species were obtained from larvae reared in our laboratory (Table A in S1 Text). For data from published descriptions, digitization was performed on the outline drawings presented or, in a few cases, on the photos of larvae (*n* = 4). Publications without a scale bar for the drawings or photos, or without upright dorsal/ ventral view, were later excluded from analysis. For reared larvae, digitization was performed on photos of larvae preserved in 30% ethanol.

Stage II larvae were chosen as the focus of this study for two reasons: 1) they sufficiently captured the among-species variations (none of the stages differed in their shape disparity, see Fig A in S1 Text); and 2) data on stage II larvae are more readily available than those on other stages in both the literature and our collection due to the difficulty of rearing to later stages. For species with differentiation of nauplius sex, female nauplii were used.

Outlines of the cephalic shield of stage II nauplii excluding the appendages were traced manually on each drawing or photo. Tracing was performed in ImageJ [16] using polygon selection followed by spline fitting for smoothing. Outlines were manually adjusted to fit the original drawing as closely as possible, and were later saved as binary masks. In addition to nauplii outlines, linear measurements of larval length, larval width, and frontal horn length (recorded as mean of the pair) were also measured. These measurements were either directly retrieved from the literature or measured from the outline drawings in ImageJ if they were not available from the source literature. Mean values of individuals within each species were used for later analysis.

### Outline processing and elliptic Fourier analysis

**Fig 1:**
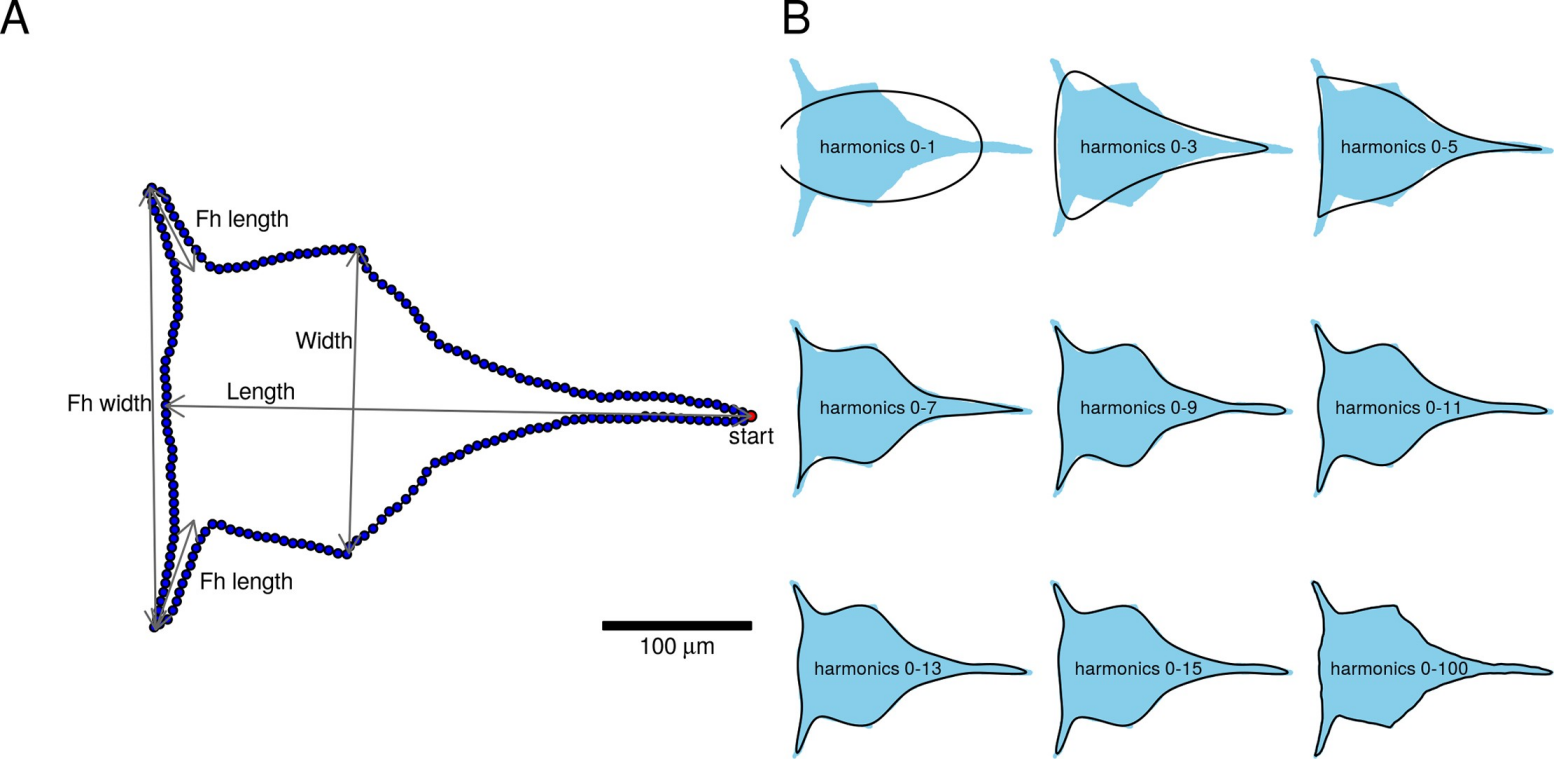
Outline processing and analysis on cephalic shield of stage II barnacle nauplius larva. (A) Shape mask labeled with the outline sampling scheme and linear measurements used. *start* denotes standardized starting point of outline sampling at tip of dorsal shield spine; *Fh* = frontal horn; *Fh width* = distance between tips of frontal horns. (B) Outline reconstructed from elliptic Fourier analysis. Blue shades are the masks of the original outline while black lines are reconstructed outlines using different number of harmonics.

A standardized number of 200 co-ordinates from the binary mask of the larvae were selected for subsequent analysis. Starting from the tip of the dorsal thoracic spine (or the most posterior point), points were sampled along the outline at regular intervals (Fig 1A).

Shape analyses were performed with normalized elliptic Fourier analyses (EFA; [17]). After normalization, the outlines were invariant to size, translation and rotation. EFA was computed according to Claude [18]. Coordinates of the outlines were described by series of harmonics functions in EFA and the coefficients of the harmonics were used in subsequent statistical analyses. The number of harmonics required to describe the outline sufficiently was usually much less than the number of points sampled, such that minute variations described by higher order harmonics were excluded to reduce data complexity. The number of harmonics used was determined by the number needed to explain > 99% of the shape data and visually inspected for a close fit between original and reconstructed outlines (Fig 1B).

### Variations in nauplii shapes

Principal component analysis (PCA) was used to explore shape variation in the nauplii. PCA was performed on the variance-covariance matrix of the shape data (i.e. coefficients of harmonics computed from the first 15 harmonics of EFA excluding the first three coefficients of the first harmonics which contain zero values after normalization). To identify the major shape changes pattern, shape changes along PC axes were visualized. To compute the hypothetical outlines on principal components, coefficients of harmonics were first calculated from the product of the PC score and its corresponding eigenvector. PC scores of mean ± 2SD on the first two principal axes were used for this calculation. Using inverse Fourier transformation, the hypothetical shapes were reconstructed from the calculated coefficients. Shape change was visualized with thin-plate spline deformation grids, shown as deformation from the overall mean outline.

Direct visualization suggested possible change in aspect ratio and relative frontal horn length (ratio of frontal horn to larval length) along the PC axes. The correlation between PC axes and changes in linear measurements of aspect ratio and relative frontal horn length were also tested. Vectors of coefficients from regression of shape data on aspect ratio and relative frontal horn length were first calculated. Then correlation (*R*) between the regression vectors and eigenvectors of PCA was calculated using the dot product of the vectors normalized by their magnitudes. We used the absolute value of *R* since the direction of PC axes are arbitrary, which give *R* value range from 0 to 1, with 0 and 1 representing orthogonal vectors and parallel vectors, respectively. Significance of *R* was determined by permutation test to test the null hypothesis of no correlation between the vectors. *R* was also expressed as an angle between the vectors with *θ* = arccos(*R*).

### Relationship between habitats, PLD and nauplii shape variations

To explore whether adult and larval habitats are important factors in determining the shape of nauplii, information on adult and larval habitat and pelagic larval duration (PLD) were incorporated into the PCA plot. Adult barnacle habitats were categorized by the type of substrate or the locations in which these substrata are found. The adult habitats were categorized as borer (Acrothoracican barnacles, which bore into calcareous substratum and live in its own created burrows), parasitic (Rhizocephalan barnacles parasitic on decapod crustaceans), deep-sea (species from hydrothermal vents and deeper waters (>200m)), inter/sub-tidal including the coastal species, epibiotic (species that live on crab carapace and crab gills) and pelagic (the genus *Lepas*, which live on floating objects). Given the dual-phasic life history of barnacles, habitats of the larvae could differ significantly from that of the adult. Larval habitat (coastal water, coral reef, deep sea and open ocean) and length of planktonic dispersal (PLD: < 5 days, 5–30 days and > 30 days) were also tested for their impact on nauplii shape.

### Effect of allometry and trophic modes on nauplii shape variations

Allometry (shape changes associated with size) and trophic mode (planktotrophy *vs*. lecithotrophy) were assessed as the factors associated with the major directions of nauplii shape change (PC1 and PC2). Allometry was assessed by regressing shape data on size (larval length). The one dimensional shape score proposed by Drake & Klingenberg [19] was calculated to check the fit of the regression between multivariate shape data and size because it is a more intuitive way to summarize the multivariate regression model. The shape score was calculated by projecting the shape data onto a line parallel to the regression vector with the equation *s* = *yβ*^T^ ⋅ (*ββ*^T^)^−0.5^, with the regression model formulated as *y* = *β***x** *+* **ε** (where *y* is the shape data; *β* is the vector of regression coefficients; *β*^T^is transposed *β***; x** is the vector of size and **ε** is the vector of residuals). To visualize the predicted shape change along the size gradient, shapes predicted by the regression model were reconstructed from the predicted values of harmonic coefficients.

To check for the association between size and PC1, correlation between the allometric regression coefficients and PC1 was tested. Similarly, correlation between regression coefficients of shape against trophic mode and PC2 was tested. To tease apart the effect of allometry and trophic mode, ‘allometry free’ shapes, which represent shape variation not explained by size variation, were computed with the residuals from the regression model. Differences in shape between trophic modes were later compared with the allometry free shapes by 1) visualizing the mean difference between trophic groups, 2) projection onto the original PCA plot and 3) statistical testing on the significance of the shape difference using trophic mode as a predictor. We used bootstrapping for statistical estimation between trophic modes (mean shapes, relative frontal horn length). Permutation tests were performed to test the significance of regression of shape on size and shape difference between trophic modes using the geomorph R package [20]. For bootstrapping, equal numbers of species were sampled for both trophic modes in each bootstrap sample (using the number of the smaller group, i.e. lecithotrophy) in an effort to correct for the unequal number of group members. Case re-sampling was used in both bootstrapping and permutation tests for consistency.

### Role of phylogeny

The phylogenetic signal of the nauplii was evaluated to examine whether barnacle nauplii shape is a function of phylogenetic relatedness. A published phylogeny was taken directly from Pérez-Losada [21], which covered a subset (*n* = 36) of the species analyzed for their shapes. Tree was digitized from the paper with TreeSnatcher plus [22]. Phylogenetic signal was estimated with generalized version of Blomberg’s *K* statistics [23] for multivariate shape data (*K*_*mult*_) [24]. Calculation of *K* statistics and assessment of significance by permutation test were carried out with the geomorph R package. *K* statistics estimate phylogenetic signal of the trait relative to what is expected under neutral evolution with the given tree (Brownian motion model), where *K* = 1 indicates that the trait’s evolution matches the model, *K* < 1 indicates that the trait are less similar among relatives than expected and *K* > 1 indicates that trait are more similar among relatives than expected. For visualization, ‘phylomorphospace’ was presented by overlaying phylogeny on the morphospace (PCA plot) of nauplii shape. If nauplii morphology carries a significant phylogenetic signal, a non-random branching pattern on the morphospace is expected. Positions of the phylogeny’s internal nodes on the morphospace were estimated by ancestral state reconstruction using the maximum likelihood method with the phytools R package [25]. In addition, we also used the phylogeny to account for non-independence among species for analyses on role of size and trophic mode with the smaller dataset where phylogeny is available. The inclusion of phylogenetic covariance in the analyses enabled us to check whether the same conclusion could be reached after accounting for the effect of common ancestry. Phylogenetic ANOVA/regression was carried out with the geomorph R package.

## Results

### Nauplii shape variations summarized by aspect ratio and relative frontal horn length

**Fig 2:**
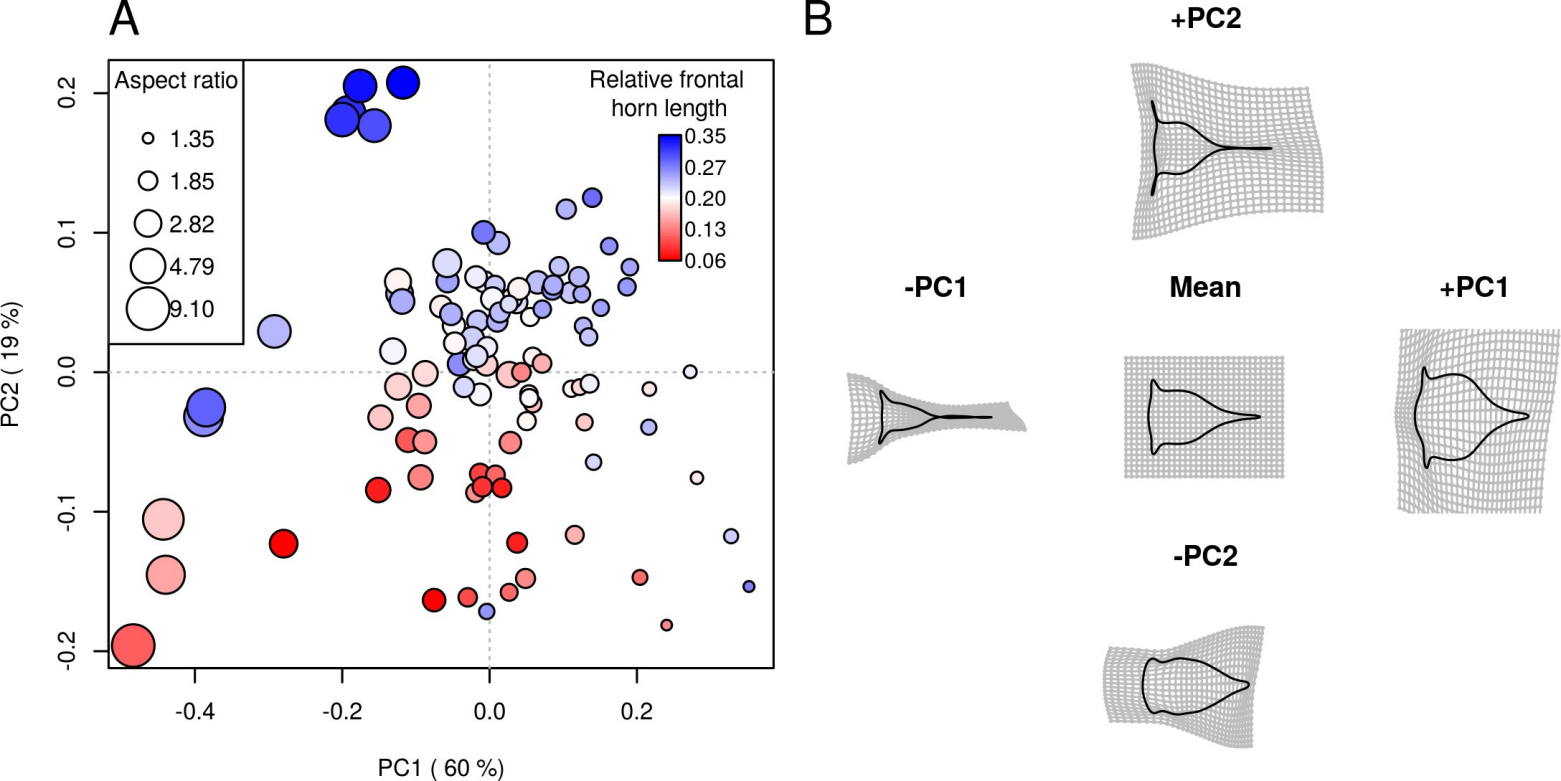
Major directions of nauplii shape variations. (A) Plot of first two principal component scores from principal component analysis (PCA) on outline shape of stage II barnacle nauplii. Data points were colored by relative frontal horn length (ratio of frontal horn length to larval length) and their sizes were scaled with larval aspect ratio (both data layers came from manual measurements). Note that scale of the color gradient and size of the aspect ratio are logarithmic. (B) Reconstructed outline shapes at ± 2SD PC score values, plotted as thin-plate-spline deformation from the mean outlines, depict the trend of shape changes along the PC axes.

Principal component analysis summarizes the overall shape variations of stage II barnacle nauplii (Fig 2). Most of the shape variations (79% in total) can be explained by the first two principal components (PCs). Most species are scattered around the centroid of this "morphospace", with a small proportion of species with extreme morphologies occupying the periphery (Fig 2A). Direct visualization of changes along the PCs shows the major directions of shape changes (Fig 2B). For PC1, nauplius shape is more rounded in the positive direction while the shape is more elongated and slender in the negative direction. For PC2, the positive direction is characterized by the long frontal horns while the negative direction shows shape with short frontal horns and rounded body with very short tail processes. Some areas in the morphospace are not occupied, i.e. the positive extreme of PC2 at either extremes of PC1. These areas correspond to 1) slender shape, long tail processes with long frontal horns and 2) rounded shape, short tail processes and long frontal horns. Observations from direct visualization prompted us to associate PC1 with change in aspect ratio and PC2 with change in relative frontal horn length. Measurements of aspect ratio and relative frontal horn length overlaid on the plot of PC scores shows trends that agree with the observations from direct visualization (Fig 2A). Permutation tests confirm that PC1 significantly correlates with aspect ratio and PC2 significantly correlates with relative frontal horn length (Table 1).

**Table 1:** Correlation (*R*) and angle (θ) between major PC axes of barnacle nauplii II morphospace and regression vectors (coefficients of regression of shape on size, aspect ratio, relative frontal horn length and trophic mode).

	*R*	θ	*p*-value
*PC & shape attributes*			
PC1 & aspect ratio (width / length)	0.992	7.0	0.001
PC1 & aspect ratio (Fh width / length)	0.948	18.5	0.061
PC2 & relative frontal horn length	0.930	21.6	0.003
*PC & traits*			
PC1 & size	0.988	8.9	0.003
PC2 & trophic mode	0.832	33.7	0.04

### Overall shape variation scales with size

**Fig 3:**
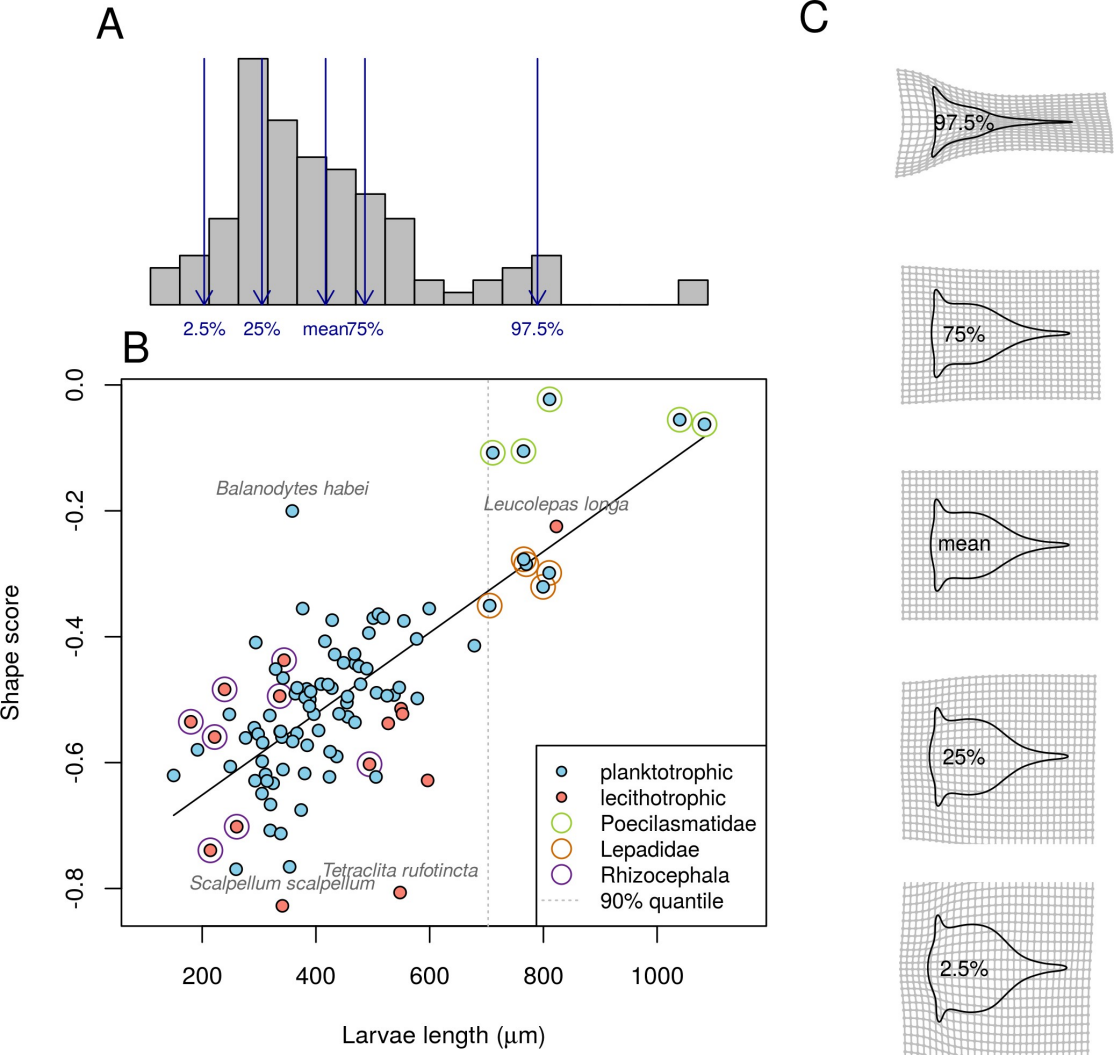
Allometry of nauplii shape. (A) Histogram of size distribution of stage II barnacle nauplii. Arrows correspond to size used for later reconstruction of predicted allometric shape changes. Dashed line indicates larval length at 90^th^ percentile for all species. (B) Shape score associated with size estimated from multivariate regression of shape of nauplii outlines on size. Species of special interest to discussion were annotated. (C) Predicted shapes from regression of shape on size at 50% and 95% intervals, shown as thin-plate-spline deformation from the predicted shape at mean size.

Larval length is a good predictor for aspect ratio (linear regression, *R*^*2*^ = 60.27%, Fig B in S1 Text). Multivariate regression of shape against size shows that larval length explains a large proportion of total variation (*R*^2^ = 30.7%, Table 2). This relationship indicates that there is a strong allometric effect on nauplii shape variation. This conclusion holds when tested with a smaller dataset for which phylogenetic non-independence was considered (Table B in S1 Text). Apermutation test on the correlation between the coefficients of this allometric regression model and PC1 is significant (Table 1), suggesting allometry as a good explaining factor for shape changes along PC1. The regression fits well linearly with no obvious problem except three possible outliers (*Balanodytes habei*, *Scalpellum scalpellum* and *Tetraclita rufotincta*, Fig 3B; regressions were also fitted with data with outliers removed, which improved the fit to a *R*^2^ of 36%, see Table C and Fig C in S1 Text). Notably, the large nauplii from Poecilasmatidae and Lepadidae, which are both >90^th^ percentile in terms of larval length, are well separated from other nauplii along the allometric shape score axis, suggesting that allometry alone is enough to differentiate them from other nauplii. Visualization of shape change along the size gradient as predicted by the regression model shows a trend of increasing aspect ratio, agreeing with the conclusion that allometry is associated with the shape changes along PC1 (Fig 3C).

**Table 2:** MANCOVA table on the effect of allometry and trophic mode on outline shapes of barnacle nauplii II. Type I (sequential) sums of squares were used in calculation.

	Df	*R*^2^	F	Z	*p*-value
size	1	0.307	52.813	19.191	0.001
trophic mode	1	0.100	17.176	7.526	0.001
size × trophic mode	1	0.023	4.000	1.776	0.087
Residuals	98				
Total	101				

### Relative frontal horn length differed between trophic modes

**Fig 4:**
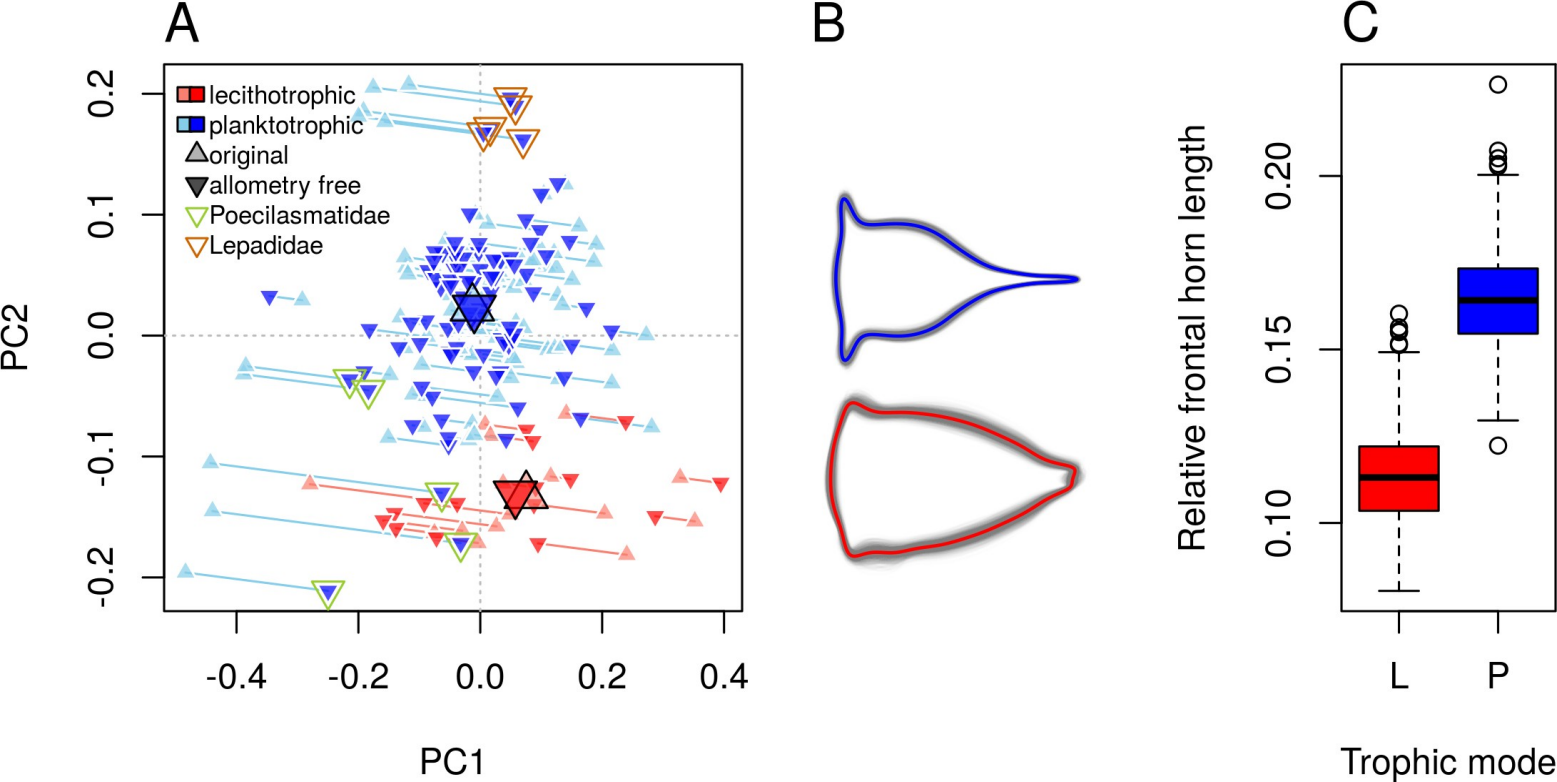
Nauplii shape differentiation between trophic modes. (A) Allometry free outlines projected back onto the original shape space as shown in Fig 2. Enlarged symbols represent the group means. Lines were drawn to connect the data points before and after adjustment for allometry, and they are parallel to the vector of size gradient (Note: the angle of these lines to PC1 is smaller than the calculated angle in Table 1 because of the distortion of high-dimensional data in 2-dimensional PC plot). (B) Bootstrapped allometry-free nauplii outlines between trophic modes reconstructed from residuals of regression of shape on size. Bootstrapped means are represented by colored outlines while the grey lines illustrates the variability of mean in the bootstrap samples. (C) Boxplot of bootstrapped means for relative frontal horn length. In all bootstrapping, *n* = 1000.

Nauplii from different trophic mode are separated along PC2 for both shapes before and after allometry correction, suggesting that: 1) trophic mode is related to shape changes along PC2, i.e. relative frontal horn length and 2) shape differentiation between the trophic modes is independent of allometry (Fig 4A; Fig D in S1 Text for adjustments with alternative methods). Permutation tests show that shape distinction between trophic modes is correlated with PC2 (Table 1) and that there is significant shape difference between planktotrophic and lecithotrophic nauplii (Table 2). This conclusion holds when tested with a smaller dataset for which phylogenetic non-independence is considered (Table B in S1 Text). However, Lepadidae are still separated from other nauplii along PC2. Visualization of mean shape difference between the trophic groups shows that planktotrophic larvae are characterized by longer frontal horns, a more slender body and a longer dorsal shield spine. Relative frontal horn length is longer in planktotrohic nauplii than lecithotrophic larvae (see Fig 4C for bootstrapped estimates with planktotrophic nauplii down-weighted during resampling to simulate a scenario of equal number of species between trophic modes, and see Fig B in S1 Text for original data).

### Phylogeny and habitat account for extreme morphologies

**Fig 5:**
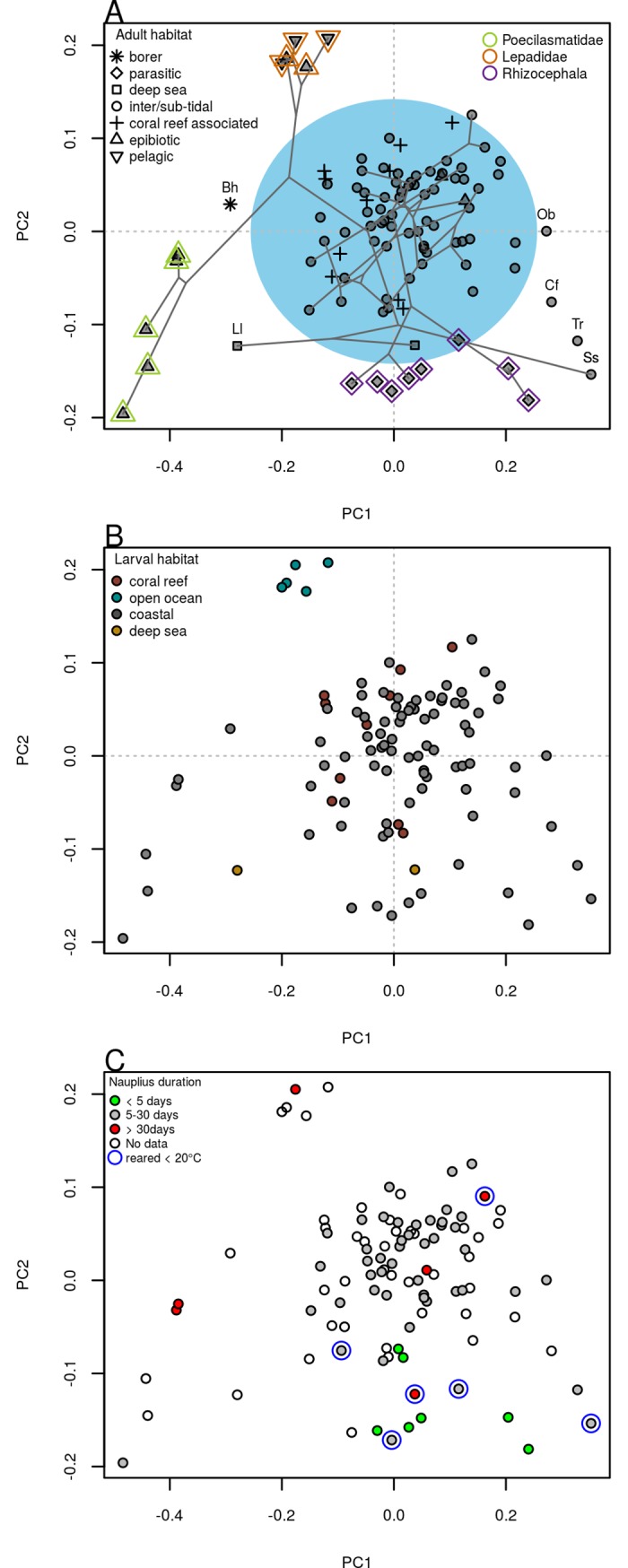
Roles of phylogeny and habitat on nauplii shape. (A) Phylomorphospace (subset of phylogeny over PCA plot from Fig 2) incorporated with information of adult habitats (Note: borer refers to Acrothoracican barnacles, which bore on calcareous substrate and live in its own created burrows; deep sea habitat refers to hydrothermal vents and open ocean >200m deep). Nauplii of most species have similar morphology and are concentrated around the center (blue shade, ~77%) while species at the periphery are more correlated with certain habitats or taxonomic groups. Peripheral species not belonging to these taxonomic groups are annotated (Bh = *Balanodytes habei*; Cf = *Chthamalus fragilis*; Ll = *Leucolepas longa*, Ob = *Octomeris brunnea*; Ss = *Scalpellum scalpellum*; Tr = *Tetraclita rufotincta*). (B) Relationship between larval habitat and nauplii shapes. Larval habitat differs from adult habitat in that there is less emphasis on substrate type. (C) Relationship between pelagic larval duration (PLD) and nauplii shape. PLD was categorized into extreme and general groups, see Fig H in S1 Text. Data for species reared at low temperature were highlighted, as low rearing temperature may greatly prolong rearing duration.

The phylomorphospace plot (i.e., overlaying the phylogenetic tree on the PCA plot) shows a random branching pattern around the center of the morphospace with lots of branch crisscrossing, while the taxa at the periphery (Poecilasmatidae, Lepadidae and Rhizocephala) follow a non-random pattern (Fig 5). Direct comparison of the phylogenetic tree with the clustering tree of nauplii shape agrees with this observation that most species have morphologies that are not tightly coupled with phylogeny (Fig E in S1 Text). In terms of the overall outline, nauplii shapes contain a significant but weak phylogenetic signal (*K*_*mult*_ = 0.42 *p* = 0.001). When the shape attributes of aspect ratio and relative frontal horn length were tested individually, aspect ratio was found to be phylogenetically conserved (*K* = 1.58, *p* = 0.001) while the relative frontal horn length has weak phylogenetic signal (*K* = 0.40, *p* = 0.02). Mapping of aspect ratio and relative frontal horn length on phylogeny is presented in Fig F in the S1 Text.

Habitat signal analysis yielded results similar to that of phylogenetic signal analysis, i.e. neither habitat nor phylogeny alone is sufficient to explain shape variation of nauplii, and this works especially poorly for the majority of species at the center of the morphospace (Fig 5A). The center of the morphospace, where most the species are located, is dominated by species associated with intertidal, subtidal and coral reef habitats. Most species with extreme morphology from pelagic, epibiotic and parasitic habitats were readily identified as being from the family Lepadidae, the family Poecilasmatidae, and the superorder Rhizocephala, respectively.

## Discussion

### Outline analysis of larvae

**Fig 6:**
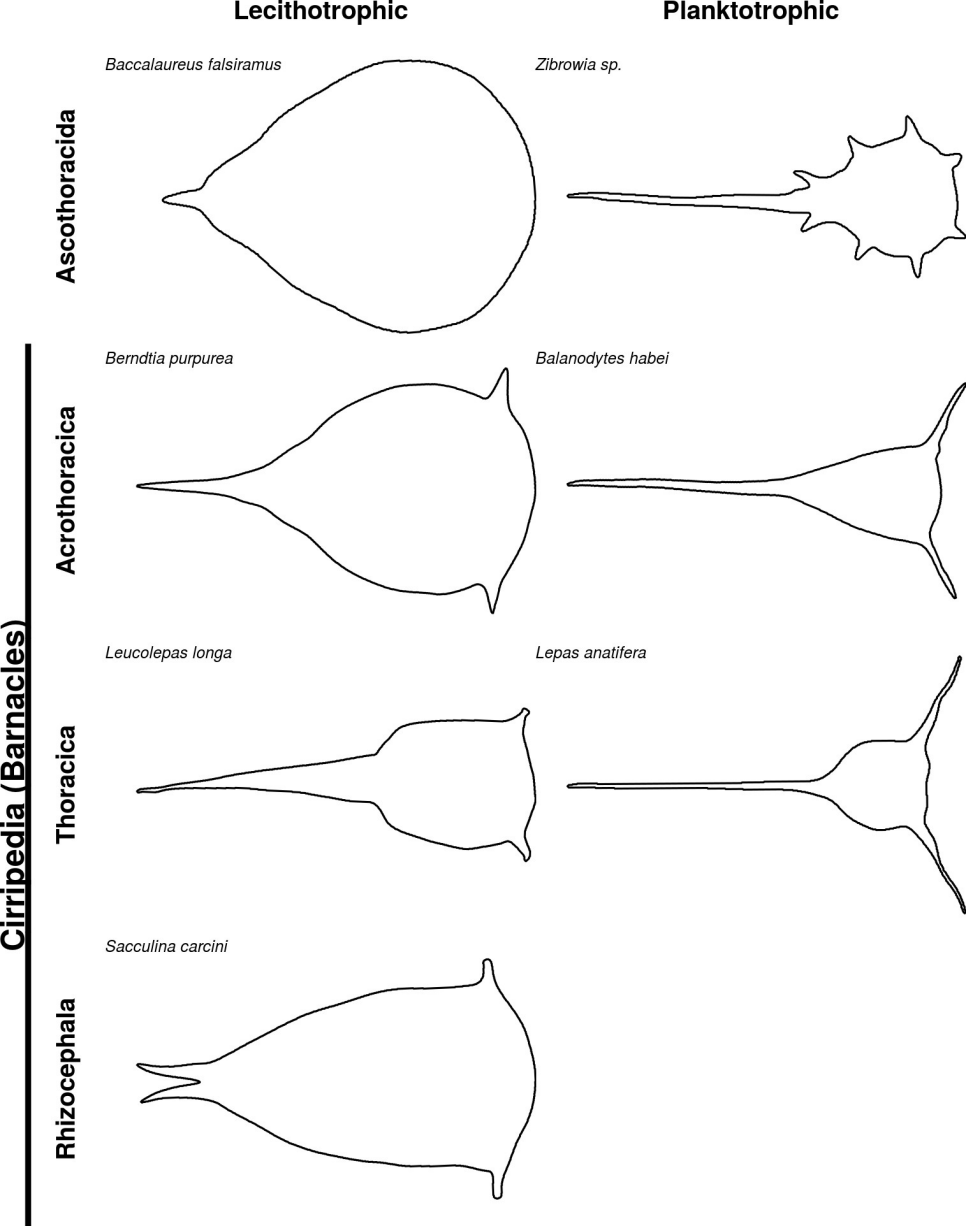
Trophic modes and naupliar morphology. Examples of naupliar cephalic shield outlines from Cirripedia (barnacles) categorized by their trophic mode. Larvae from Ascothoracida, the Thecostracan sister group to Cirripedia, were also included (outlines traced from photos in [46]). Frontal horns are only present in barnacle nauplii and are absent in other crustacean nauplii. Relative frontal horn length is likely related to feeding.

We used the geometric morphometrics method to provide an updated summary and analysis on the morphological variations of the barnacle naupliar cephalic shield. Outline analysis is sometimes referred to as the "landmark-free" morphometrics method, and is potentially useful for comparisons between taxa involving the gain or loss of character [15] (but also see [26]). For example, it is difficult to compare barnacle nauplii with other crustacean nauplii without frontal horns in a morphometrics study (see example of Ascothoracida in Fig 6). Outline analysis also allows the inclusion of more detailed shape variations, which may increase the effect sizes on shape differences [27]. However, the homology-free property of outline analysis also poses challenges to properly aligning outlines and to interpreting observed variations [28]. Another limitation of this analysis approach is that barnacle nauplii outlines are particularly susceptible to the problem of the “Pinocchio effect” [29] because of the large variations in the length of tail processes. Despite these limitations, when coupled with visualization of shape changes, outline analysis can provide an intuitive way to identify important patterns and summarize morphological changes for large numbers of species. Furthermore, output from these analyses can be used to inform computational fluid dynamics or dynamically scaled physical models. For instance, mean shapes can be used as morphology representative for all species of interest, and predicted shapes resulting from a regression model can be used for hypothesis testing concerning specific factors of interest.

### Functional constraints imposed by swimming and feeding

Our results suggest that adaptation to adult habitats alone does not account for overall nauplii shape variation. Allometry and trophic mode, however, provide better explanations for the overall nauplii shape variations. In many animals, aspect ratio is a function of size and dominates the major direction of shape variations in various anatomical structures: e.g. fish body form [30], bird beaks [31] and mammal skulls [32]. Such a change in aspect ratio as a function of size is due to a combination of developmental and functional constraints [33]. A shared developmental program restricts the range of variations. However, within such developmental constraints, species can easily “explore” this specified range, possibly facilitating a rapid response to selection [34]. Birds’ beak illustrated how functional constraints limit aspect ratio as it is correlated with the load the beak can take without risking fracture [35]. For barnacle nauplii, little is known about the developmental constraint on body form, but there is likely a functional constraint related to swimming. Hydrodynamic drag increases with larval size, and a higher aspect ratio can lower drag [36]. To swim, the propulsive force has to be higher than the drag, thus species with larger nauplii may be selected to have a higher aspect ratio.

Lecithotrophy repeatedly evolved within many phyla of marine invertebrates and is often accompanied by similar simplification of morphology, showing a high degree of convergence [36]. Regardless of the superorder to which the lecithotrophic nauplii belong to (Rhizocephala, Thoracica or Acrothoracica) or the habitat they originate from (deep sea, coral reef associated, parasitic or intertidal), they all occupy the same space morphospace, suggesting convergence (definition *sensu* Stayton [37]) of nauplii shape. Reduced body form in lecithotrophic marine invertebrate larvae has long been described [36], but not quantified. Our approach quantitatively shows that lecithotrophic nauplii have a reduced body form characterized by shorter frontal horns and tail processes, and a more rounded body (Fig 4).

In addition to the difference in relative frontal horn length between trophic modes, considerable variations were also observed between planktotrophic nauplii. In particular, Lepadidae nauplii have very long frontal horns. One plausible hypothesis is that frontal horns help increase hydrodynamic drags, which increase feeding efficiency of small planktons by acting as partial tether [38]. However, the role of frontal horns in feeding has been contested as this character is present in both planktotrophic and lecithotrophic larvae [2]. If the transition to lecithotrophy occurred faster than the accompanied changes in morphology [39], rudimentary structures used for feeding in planktotrophic larvae could have been retained [40]. However, some taxa with lecithotrophic nauplii, such as the Rhizocephalan barnacles, diverged long time ago (625–596 mya [41]) and were likely to have ample time to lose the structure. Alternatively, frontal horns could serve other function(s) not unrelated to feeding that are retained in lecithotrophic nauplii. Frontal horns have gland cells, which are retained in lecithotrophic nauplii and also in the non-feeding cyprids [42]. The unknown function of the gland cells might have prevented the loss of the associated physical structure of frontal horns, perhaps due to linkages in developmental program. Interestingly, the presence of long frontal horns may also disrupt flow around larval bodies creating small eddies which could contribute towards chemosensing. The hydromechanical function of this unique morphological feature warrants further investigations.

Given both developmental and functional constraints, observed morphologies should reflect the outcome of functional trade-offs. Nauplii with very long frontal horns together with a very high or low aspect ratio are not observed, possibly because swimming ability could not be maintained with such morphology. Functional trade-offs in nauplii shape may have caused the clustering at the center of the morphospace with majority of species displaying a ‘generalized’, possibly ‘compromised’ shape [7]. Morphospace pattern with a species rich clade packing the center of the morphospace has been reported for other animals such as fishes and bivalves [43, 44], and stabilizing selection due to functional constraint has been proposed as one of the factors driving such a pattern [45].

### Extreme morphology and the role of habitat

Habitats of the sessile adults have a less direct influence on barnacle planktonic larvae; therefore, it is not surprising that barnacle naupliar morphology generally does not change with adult habitats. Our results partially support the idea that larval morphology is not tightly related to adult habitat as is often assumed [47] and that the inter-specific morphological diversity is achieved in post larval stages [48].

Larvae with extreme morphology—i.e. those occupying the periphery of the morphospace—are from the family Lepadidae, the family Poecilasmatidae, and the superorder Rhizocephala. The observed phylogenetic signal is likely a reflection of habitat-associated selective pressure exerted over the species at some part of their evolutionary history.

For Lepadidae species, their nauplii possess complex setation and large labrum to capture sparse food particles in the open ocean [6, 7]. This morphological change is distinct from other groups for which feeding was lost and larval morphology became “simplified” (see discussion above): e.g. *Leucolepas longa* and *Neoverucca* sp. found in deep-sea hydrothermal vents and coastal *Tetraclita rufotincta* which reproduce during the food-limited winter.

For nauplii of Poecilasmatidae and Rhizocephala, the substrate type of their adults (epibiotic and parasitic) might play a more important role in shaping their overall form. Scarcity of settlement substrate could select for extreme pelagic larval duration: long larval duration (Poecilasmatidae) may enhance chances for opportunistic encounter with substrate, while short larval duration (Rhizocephala) may enhance chances for settling in favorable substrate near the parent. We observed a positive relationship between extremely long PLD and extremely large larval size (Fig 5C; Fig G in S1 Text), which could suggest that increased larval size favors long distance dispersal and small size favors rapid development. This association could also explain why other species from epibiotic habitat, such as *Chelonibia testudinaria* and *Coronula diadema*, do not display extreme shape as they do not possess extreme larval duration.

As evolution progresses, yesterday’s adaptation may become the present day’s constraint [49]. Given that morphology is confounded by phylogenetic relatedness, we observed high phylogenetic signal in species with extreme morphology. The scenario discussed here only represents one of the many possibilities, and future studies on the evolution of barnacle larval morphology will benefit from modeling studies that explicitly evaluate all possible evolutionary models and clarify the relations among the inter-related factors of habitat, phylogeny, development and functional constraints.

## Conclusions

Of the 102 species surveyed, the overall morphology of barnacle naupliar larvae shared a high degree of similarity suggesting size-related biomechanical or developmental constraints, such that feeding and other functional requirements are important in shaping the evolution of larval form. Larvae from family Lepadidae, family Poecilasmatidae, and superorder Rhizocephala, however, have extreme morphologies with increased aspect ratio and/or frontal horn length. These extreme characters are likely associated with their larval ecology: scarcity of food or settlement habitats. Quantitatively summarizing variations in larval morphology and testing their link to phylogeny and ecology help better inform evolutionary constraints and understand functional morphology of an important life stage.

## Supporting information

S1 Text Supplementary tables, figures and references. (DOCX)

## References

[1] Lai JCY, Mendoza JCE, Guinot D, Clark PF, Ng PKL. Zool Anz. 2011;250(4):407–448.

[2] Høeg JT, Møller OS. When similar beginnings lead to different ends: constraints and diversity in cirripede larval development. Invertebr Reprod Dev. 2006;49(3):125–142.

[3] Clay TW, Grünbaum D. Swimming performance as a constraint on larval morphology in plutei. Mar Ecol Prog Ser. 2011;423:185–96.

[4] Walker G. Swimming speeds of the larval stages of the parasitic barnacle, *Heterosaccus lunatus* (Crustacea: Cirripedia: Rhizocephala). J Mar Biol Assoc U.K. 2004;84(4):737–742. 10.1016/s0022-0981(00)00284-7 11077064

[5] Olesen J. The crustacean carapace—morphology, function, development, and phylogenetic history In: Watling L, Thiel M, editors. Functional morphology and diversity. New York: Oxford University Press; 2012 p. 103–139.

[6] Moyse J. Some observations on the swimming and feeding of the nauplius larvae of *Lepas pectinata* (Cirripedia: Crustacea). Zool J Linn Soc. 1984;80(2–3):323–336.

[7] Moyse J. Larvae of lepadomorph barnacles In: Southward AJ, editor. Barnacle biology. No. 5 in Crustacean Issues. Netherlands: A. A. Balkema; 1987 p. 329–362.

[8] Chan BKK, Garm A, Høeg JT. Setal morphology and cirral setation of thoracican barnacle cirri: adaptations and implications for thoracican evolution. J Zool. 2008;275(3):294–306.

[9] Marchinko KB, Palmer AR. Feeding in flow extremes: dependence of cirrus form on wave-exposure in four barnacle species. Zool. 2003;106(2):127–141. 10.1078/0944-2006-00107 16351898

[10] Trager GC, Hwang JS, Strickler JR. Barnacle suspension-feeding in variable flow. Mar Biol. 1990;105(1):117–127.

[11] Miller KM, Roughgarden J. Descriptions of the larvae of *Tetraclita rubescens* and *Megabalanus californicus* with a comparison of the common barnacle larvae of the Central California coast. J Crust Biol. 1994;14(3):579–600.

[12] Ross PM, Burrows MT, Hawkins SJ, Southward AJ, Ryan KP. A key for the identification of the nauplii of common barnacles of the British Isles, with emphasis on *Chthamalus*. J Crust Biol. 2003;23(2):328–340.

[13] Lee C, Shim JM, Kim CH. Larval development of *Capitulum mitella* (Cirripedia: Pedunculata) reared in the laboratory. J Mar Biol Assoc U.K. 2000;80(03):457–464.

[14] Chan KYK. Biomechanics of larval morphology affect swimming: insights from the sand dollars *Dendraster excentricus*. Integr Comp Biol. 2012;52(4):458–469. 10.1093/icb/ics092 22753391

[15] Polly PD. Developmental dynamics and G-matrices: can morphometric spaces be used to model phenotypic evolution? Evol Biol. 2008;35(2):83–96.

[16] Schneider CA, Rasband WS, Eliceiri KW. NIH Image to ImageJ: 25 years of image analysis. Nat Methods. 2012;9(7):671–675. 22930834 10.1038/nmeth.2089 PMC5554542

[17] Rohlf FJ, Archie JW. A comparison of Fourier methods for the description of wing shape in mosquitoes (Diptera: Culicidae). Syst Biol. 1984;33(3):302–317.

[18] Claude J. Morphometrics with R New York: Springer; 2008.

[19] Drake AG, Klingenberg CP. The pace of morphological change: historical transformation of skull shape in St Bernard dogs. Proc Biol Sci. 2008;275(1630):71–76. 10.1098/rspb.2007.1169 17956847 PMC2562403

[20] Adams DC, Otárola-Castillo E. geomorph: an R package for the collection and analysis of geometric morphometric shape data. Methods Ecol Evol. 2013;4(4):393–399.

[21] Pérez-Losada M, Harp M, Høeg JT, Achituv Y, Jones D, Watanabe H, et al. The tempo and mode of barnacle evolution. Mol Phylogenet Evol. 2008;46(1):328–346. 10.1016/j.ympev.2007.10.004 18032070

[22] Laubach T, von Haeseler A, Lercher MJ. TreeSnatcher plus: capturing phylogenetic trees from images. BMC Bioinformatics. 2012;13(1):110. 22624611 10.1186/1471-2105-13-110 PMC3411374

[23] Blomberg SP, Garland T, Ives AR. Testing for phylogenetic signal in comparative data: behavioral traits are more labile. Evolution. 2003;57(4):717–745. 12778543 10.1111/j.0014-3820.2003.tb00285.x

[24] Adams DC. A generalized *K* statistic for estimating phylogenetic signal from shape and other high-dimensional multivariate data. Syst Biol. 2014;63(5):685–697. 10.1093/sysbio/syu030 24789073

[25] Revell LJ. phytools: an R package for phylogenetic comparative biology (and other things). Methods Ecol and Evol. 2012;3(2):217–223.

[26] Klingenberg CP. Novelty and “homology-free” morphometrics: what’s in a name? Evol Biol. 2008;35(3):186–190.

[27] Collyer ML, Sekora DJ, Adams DC. A method for analysis of phenotypic change for phenotypes described by high-dimensional data. Heredity. 2015;115:357–365. 10.1038/hdy.2014.75 25204302 PMC4815463

[28] McCane B. Shape variation in outline shapes. Syst Biol. 2013;62(1):134–146. 10.1093/sysbio/sys080 22993142

[29] Klingenberg CP. Visualizations in geometric morphometrics: how to read and how to make graphs showing shape changes. Hystrix. 2013;24(1):15–24.

[30] Claverie T, Wainwright PC. A morphospace for reef fishes: elongation is the dominant axis of body shape evolution. PLoS ONE. 2014;9(11):1–11. 10.1371/journal.pone.0112732 PMC4237352 25409027

[31] Bright JA, Marugán-Lobón J, Cobb SN, Rayfield EJ. The shapes of bird beaks are highly controlled by nondietary factors. Proc Natl Acad Sci U S A. 2016;113(19):5352–5357. 10.1073/pnas.1602683113 27125856 PMC4868483

[32] Cardini A, Polly PD. Larger mammals have longer faces because of size-related constraints on skull form. Nat Commun. 2013;4(2458). 10.1038/ncomms3458 24045342

[33] Fritz JA, Brancale J, Tokita M, Burns KJ, Hawkins MB, Abzhanov A, et al Shared developmental programme strongly constrains beak shape diversity in songbirds. Nat Commun. 2014;5(3700). 10.1038/ncomms4700 24739280

[34] Renaud S, Auffray JC. The direction of main phenotypic variance as a channel to evolution: cases in murine rodents. Hystrix. 2013;24(1):85–93.

[35] Soons J, Genbrugge A, Podos J, Adriaens D, Aerts P, Dirckx J, et al Is beak morphology in Darwin’s finches tuned to loading demands? PLoS ONE. 2015;10(6):1–14. 10.1371/journal.pone.0129479 PMC4466803 26068929

[36] Emlet RB. Body form and patterns of ciliation in nonfeeding larvae of echinoderms: functional solutions to swimming in the plankton? Am Zool. 1994;34(4):570–585.

[37] Stayton CT. The definition, recognition, and interpretation of convergent evolution, and two new measures for quantifying and assessing the significance of convergence. Evolution. 2015;69(8):2140–2153. 10.1111/evo.12729 26177938

[38] Emlet RB, Strathman RR. Gravity, drag, and feeding currents of small zooplankton. Science. 1985;228(4702):1016–1017. 10.1126/science.228.4702.1016 17797666

[39] McEdward LR, Janies DA. Relationships among development, ecology, and morphology in the evolution of Echinoderm larvae and life cycles. Biol J Linn Soc Lond. 1997;60(3):381–400.

[40] Pernet B. Persistent ancestral feeding structures in nonfeeding annelid larvae. Biol Bull. 2003;205(3):295–307. 10.2307/1543293 14672984

[41] Pérez-Losada M, Høeg JT, Crandall KA. Unraveling the evolutionary radiation of the thoracican barnacles using molecular and morphological evidence: a comparison of several divergence time estimation approaches. Syst Biol. 2004;53(2):244–64. 10.1080/10635150490423458 15205051

[42] Walker G. Frontal horns and associated gland cells of the nauplii of the barnacles, *Balanus hameri*, *Balanus balanoides* and *Elminius modestus* (Crustacea: Cirripedia). J Mar Biol Assoc U.K. 1973;53(2):455–463.

[43] Price SA, Claverie T, Near TJ, Wainwright PC. Phylogenetic insights into the history and diversification of fishes on reefs. Coral Reefs. 2015;34(4):997–1009.

[44] Huang S, Roy K, Jablonski D. Origins, bottlenecks, and present-day diversity: Patterns of morphospace occupation in marine bivalves. Evolution. 2015;69(3):735–746. 10.1111/evo.12608 25611893

[45] Roelants K, Haas A, Bossuyt F. Anuran radiations and the evolution of tadpole morphospace. Proc Natl Acad Sci U S A. 2011;108(21):8731–8736. 10.1073/pnas.1100633108 21555583 PMC3102353

[46] Høeg JT, Chan BKK, Kolbasov GA, Grygier MJ. Ascothoracida In: Martin JW, Olesen J, Høeg JT, editors. Atlas of crustacean larvae. Baltimore: John Hopkins University Press; 2014 p. 104–106.

[47] Gignac P, Santana S. A bigger picture: organismal function at the nexus of development, ecology, and evolution: an introduction to the symposium. Integr Comp Biol. 2016;56(3):369–372. 10.1093/icb/icw080 27413091

[48] Katz HR, Hale ME. A large-scale pattern of ontogenetic shape change in ray-finned fishes. PLoS ONE. 2016;11(3):1–7. 10.1371/journal.pone.0150841 PMC4778928 26943126

[49] Blomberg SP, Garland T. Tempo and mode in evolution: phylogenetic inertia, adaptation and comparative methods. J Evol Biol. 2002;15(6):899–910.

