# Supplementary material for: Phylogenetic, ecological and biomechanical constraints on larval form: A comparative morphological analysis of barnacle nauplii
Source: PLoS One. 2018 Nov 8;13(11):e0206973. doi: 10.1371/journal.pone.0206973 (PMC6224274; doi:10.1371/journal.pone.0206973)
Supplement: S1 Text — (DOCX) [file pone.0206973.s001.docx]

## S1 Text

### Supplementary tables

**Table A:** List of species used in this study

| **Species** | **Reference** |
| --- | --- |
| *Acasta cyathus* | This study |
| *Acasta spongites* | [1] |
| *Adna anglica* | [1] |
| *Amphibalanus amphitrite* | [2] |
| *Amphibalanus eburneus* | [3] |
| *Amphibalanus improvisus* | [4] |
| *Amphibalanus reticulatus* | [5] |
| *Amphibalanus subalbidus* | [6] |
| *Amphibalanus variegatus* | [2] |
| *Amphibalanus venustus* | [6] |
| *Austrobalanus imperator* | [7] |
| *Austromegabalanus nigrescens* | [8] |
| *Austrominius convertus* | [9] |
| *Austrominius modestus* | [10] |
| *Balanodytes habei* | [11] |
| *Balanus balanus* | [12] |
| *Balanus crenatus* | [13] |
| *Balanus glandula* | [14] |
| *Balanus nubilus* | [15] |
| *Balanus spongicola* | [16] |
| *Balanus trigonus* | [6] |
| *Berndtia purpurea* | This study |
| *Berndtia utinomii* | This study |
| *Briarosaccus callosus* | [17] |
| *Briarosaccus tenellus* | [18] |
| *Capitulum mitella* | This study |
| *Catomerus polymerus* | [19] |
| *Chamaesipho brunnea* | [10] |
| *Chamaesipho columna* | [10] |
| *Chamaesipho tasmanica* | [19] |
| *Chelonibia testudinaria* | [20] |
| *Chthamalus antennatus* | [19] |
| *Chthamalus challengeri* | [21] |
| *Chthamalus dentatus* | [22] |
| *Chthamalus fragilis* | [6] |
| *Chthamalus malayensis* | [23] |
| *Chthamalus montagui* | [24] |
| *Chthamalus neglectus* | [25] |
| *Chthamalus stellatus* | [24] |
| *Conchoderma auritum* | [26] |
| *Conchoderma virgatum* | This study |
| *Conopea galeatus* | [27] |
| *Coronula diadema* | [28] |
| *Darwiniella angularis* | This study |
| *Epopella plicata* | [10] |
| *Euacasta dofleini* | This study |
| *Euraphia pilsbryi* | [21] |
| *Fistulobalanus albicostatus* | [29] |
| *Fistulobalanus kondakovi* | [30] |
| *Fistulobalanus pallidus* | [31] |
| *Galkinius tabulatus* | This study |
| *Hesperibalanus fallax* | [32] |
| *Hesperibalanus hesperius* | [33] |
| *Heterosaccus lunatus* | [34] |
| *Heterosaccus papillosus* | [35] |
| *Hexaminius popeiana* | [9] |
| *Jehlius cirratus* | [36] |
| *Lepas anatifera* | [37] |
| *Lepas anserifera* | This study |
| *Lepas pectinata* | [6] |
| *Leucolepas longa* | [38] |
| *Lithotrya dorsalis* | [39] |
| *Megabalanus californicus* | [40] |
| *Megabalanus rosa* | [41] |
| *Megabalanus volcano* | [41] |
| *Membranobalanus koreanus* | [42] |
| *Microeuraphia aestuarii* | [43] |
| *Microeuraphia withersi* | [44] |
| *Neotrevathana elongatum* | [45] |
| *Neoverruca sp.* | [46] |
| *Notochthamalus scabrosus* | [37] |
| *Notomegabalanus algicola* | [32] |
| *Octolasmis angulata* | [47] |
| *Octolasmis cor* | [47] |
| *Octolasmis forresti* | [6] |
| *Octolasmis lowei* | [48] |
| *Octolasmis warwicki* | This study |
| *Octomeris angulosa* | [32] |
| *Octomeris brunnea* | [49] |
| *Pollicipes pollicipes* | [50] |
| *Pollicipes polymerus* | [51] |
| *Polyascus plana* | [52] |
| *Polyascus polygenea* | [53] |
| *Pseudoctomeris sulcata* | [54] |
| *Sacculina carcini* | [55] |
| *Sacculina pilosella* | [56] |
| *Scalpellum scalpellum* | [57] |
| *Semibalanus balanoides* | [58] |
| *Semibalanus cariosus* | [13] |
| *Striatobalanus cristatus* | [59] |
| *Tesseropora pacifica* | [60] |
| *Tesseropora rosea* | [7] |
| *Tetraclita japonica formosana* | This study |
| *Tetraclita japonica japonica* | This study |
| *Tetraclita kuroshioensis* | This study |
| *Tetraclita rubescens* | [40] |
| *Tetraclita rufotincta* | [61] |
| *Tetraclita serrata* | [34] |
| *Tetraclita squamosa* | [62] |
| *Tetraclitella karandei* | [63] |
| *Tetraclitella purpurascens* | [7] |
| *Verruca stroemia* | [58] |

**Table B:** MANCOVA table of effect of allometry and trophic mode on outline shapes of subset of barnacle nauplii II species (*n* = 36). Comparison was made between MANCOVA models after and before inclusion of phylogenetic covariance. Interaction is not significant in either models and thus not included

|  | **Df** | ***R*^2^** | **F** | **Z** | ***p*-value** |
| --- | --- | --- | --- | --- | --- |
| *Adjusted for phylogeny* |  |  |  |  |  |
| size | 1 | 0.201 | 8.559 | 5.270 | 0.002 |
| trophic mode | 1 | 0.022 | 0.940 | 8.087 | 0.001 |
| Residuals | 33 |  |  |  |  |
| Total | 35 |  |  |  |  |
|  |  |  |  |  |  |
| *Without adjustment* |  |  |  |  |  |
| size | 1 | 0.415 | 27.659 | 10.889 | 0.001 |
| trophic mode | 1 | 0.090 | 6.001 | 2.440 | 0.023 |
| Residuals | 33 |  |  |  |  |
| Total | 35 |  |  |  |  |

**Table C:** MANCOVA table of effect of allometry and trophic mode on outline shapes of barnacle nauplii II with outliers removed

|  | **Df** | ***R*^2^** | **F** | **Z** | ***p*-value** |
| --- | --- | --- | --- | --- | --- |
| size | 1 | 0.360 | 64.087 | 19.769 | 0.001 |
| trophic mode | 1 | 0.084 | 14.931 | 6.234 | 0.001 |
| Size × trophic mode | 1 | 0.023 | 4.134 | 1.721 | 0.090 |
| Residuals | 95 |  |  |  |  |
| Total | 98 |  |  |  |  |

### Supplementary figures

**
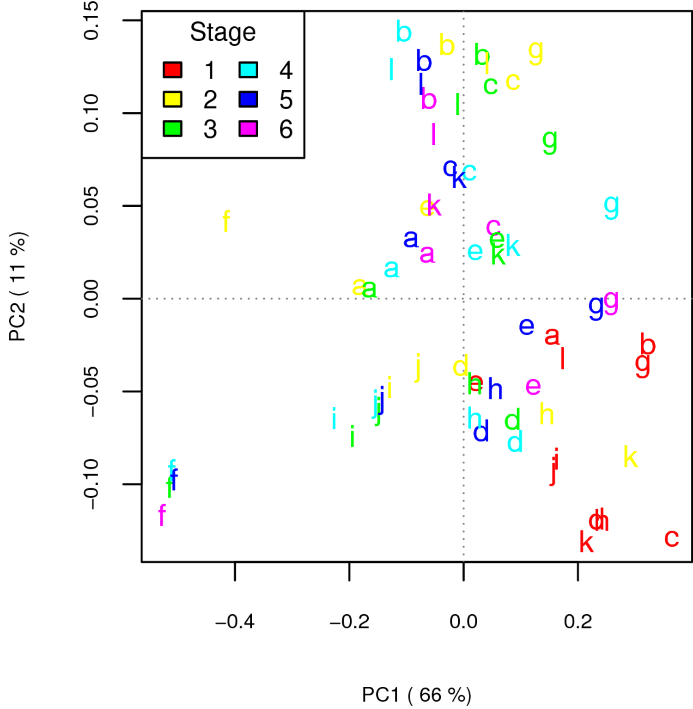
Fig. A:** **Nauplii shape variations across developmental stages**. Plot of principal component analysis of the barnacle nauplii outlines for 12 species across different developmental stages. There is significant difference in shape between stage I and all others, but no difference between any stages from stage II to VI. For morphological disparity, there is no significant difference among all stages. a: *Balanus balanus*; b: *Catomerus polymerus*; c: *Chelonibia testudinaria*; d: *Heterosaccus papillosus*; e: *Jehlius cirratus*; f: *Octolasmis cor*; g: *Pollicipes polymerus*; h: *Polyascus polygenea*; i: *Sacculina carcini*; j: *Sacculina pilosella*; k: *Tetraclita rufotincta*; l: *Tetraclitella purpurascens*


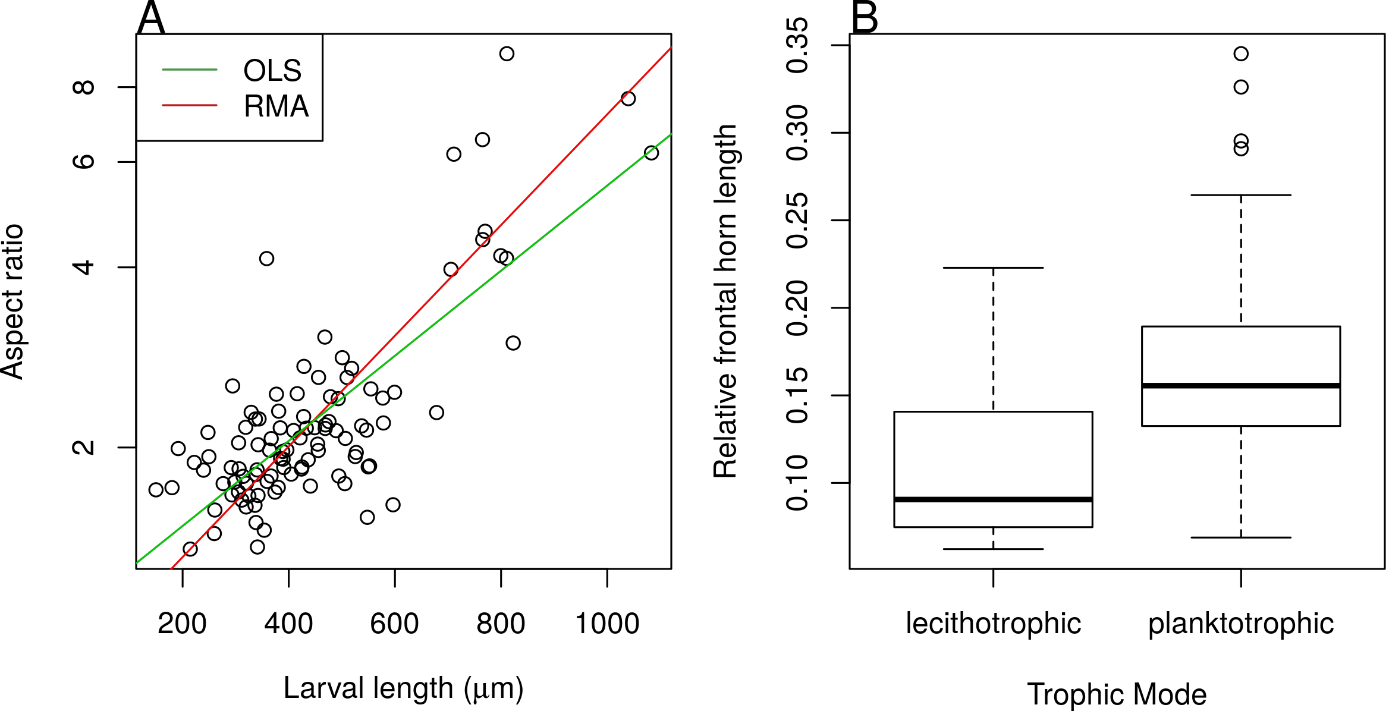


**Fig. B:** (**A**) Regression of larval aspect ratio on larval length. For both models, *R^2^* = 60.27% with a *p*-value of 0.001. Regression lines were shown with two different regression method: OLS = Ordinary Least Square and RMA = Range Major Axis. Note the logarithmic scale of y-axis. (**B**) Relative frontal horn length by trophic modes. The four outliers for planktotrophic nauplii are *Conchoderma auritum*, *Lepas anatifera*, *Lepas anserifera* and *Lepas pectinata*.


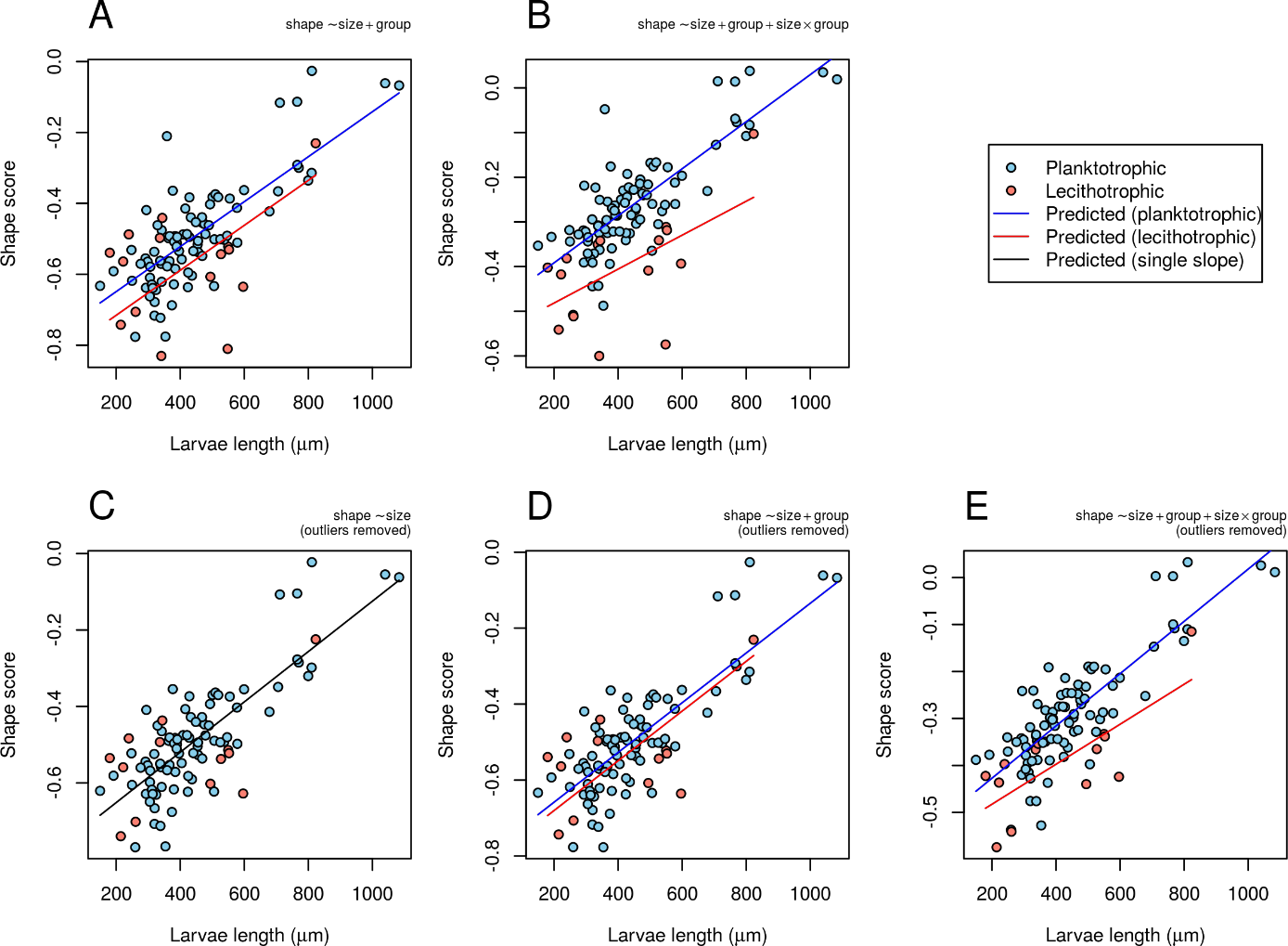


**Fig. C:** **Regression scores from different models of regression of shape on size.** (**A**, **D**) Common slope different intercept model. (**B**, **E**) Different slopes model. (**C**) Single slope model. (**C**, **D**, **E**) Single slope model, common slope different intercept model and different slopes model after removal of outliers.


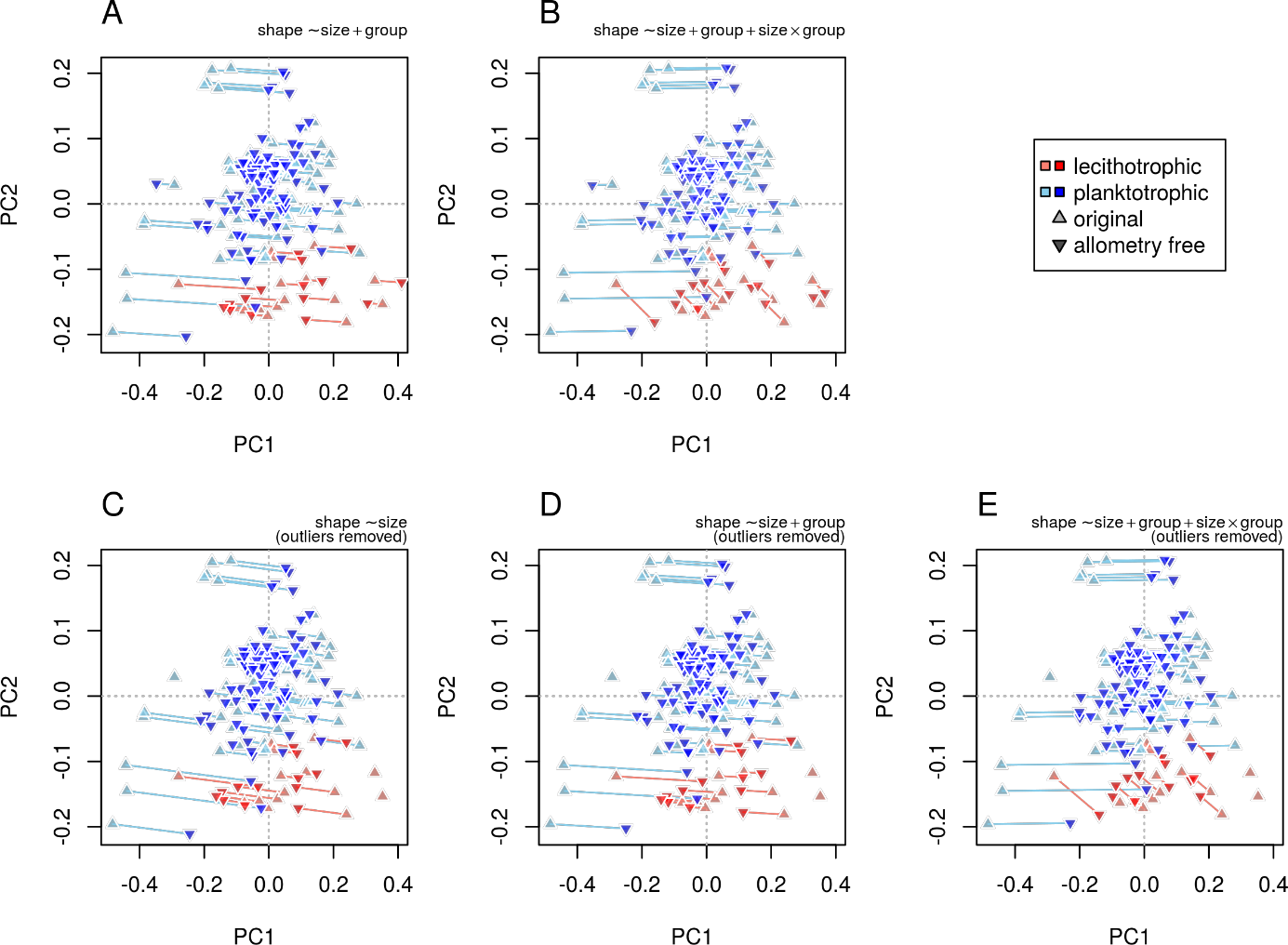


**Fig. D:** **Allometry adjustment projected back onto original PCA plot with different allometric regression models**: (**A**, **D**) Common slope different intercept model. (**B**, **E**) Different slopes model. (**C**) Single slope model. (**C**, **D**, **E**) Single slope model, common slope different intercept model and different slopes model after removal of outliers.


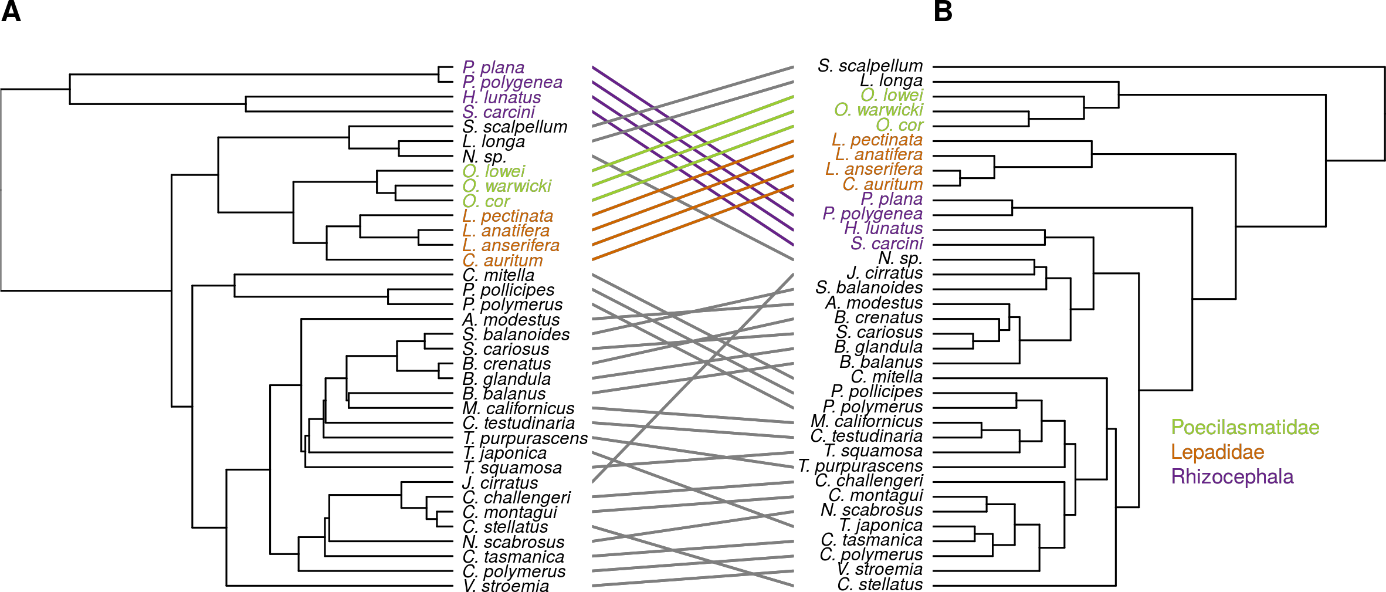


**Fig. E:** **Comparison between trees inferred from molecular phylogeny and nauplii body form.** (**A**) Maximum likelihood phylogeny from [64]. Note the tree was made ultrametric here. (**B**) UPGMA hierarchical clustering of nauplii shapes. Optimal disentangling of the connections between the trees was computed with the cophylo function from the phytools package [65]. Refer to Fig. F for full species names.


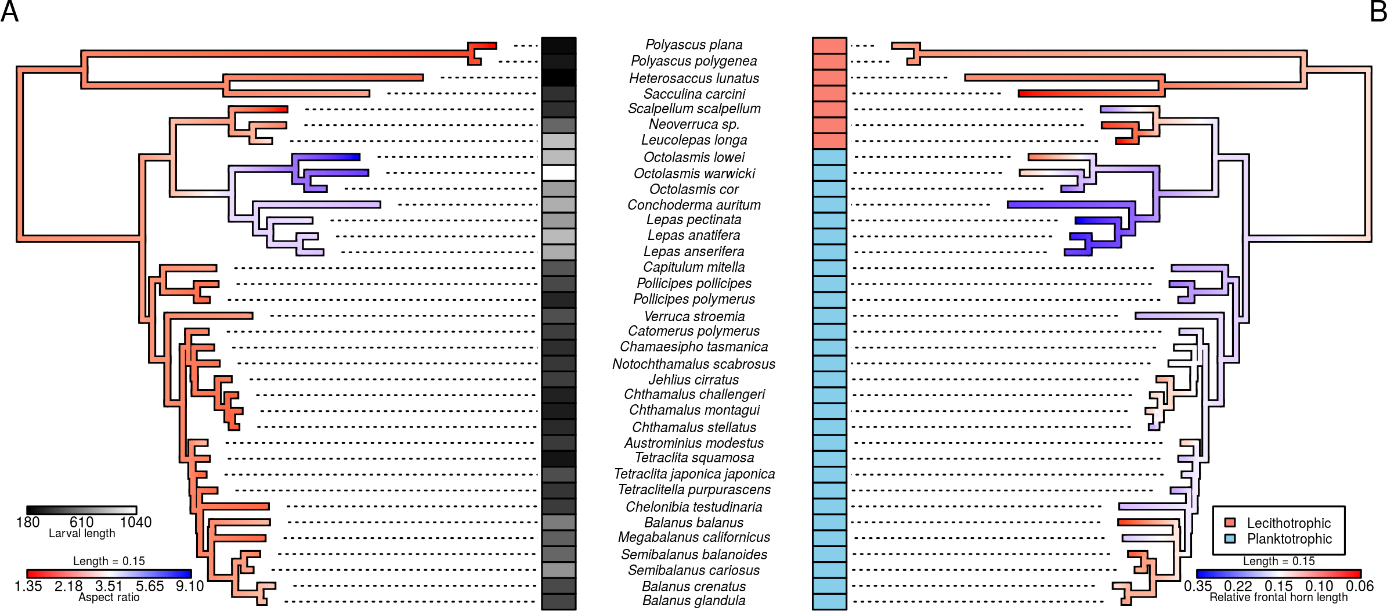


**Fig. F:** **Larval morphological traits mapped onto phylogeny and their relation to size and trophic mode.** (**A**) Aspect ratio mapped onto phylogeny. Ancestral state was estimated by maximum likelihood method. Variation in size is shown by side. (**B**) Relative frontal horn length mapped onto phylogeny. Distinction of trophic mode is shown by side.


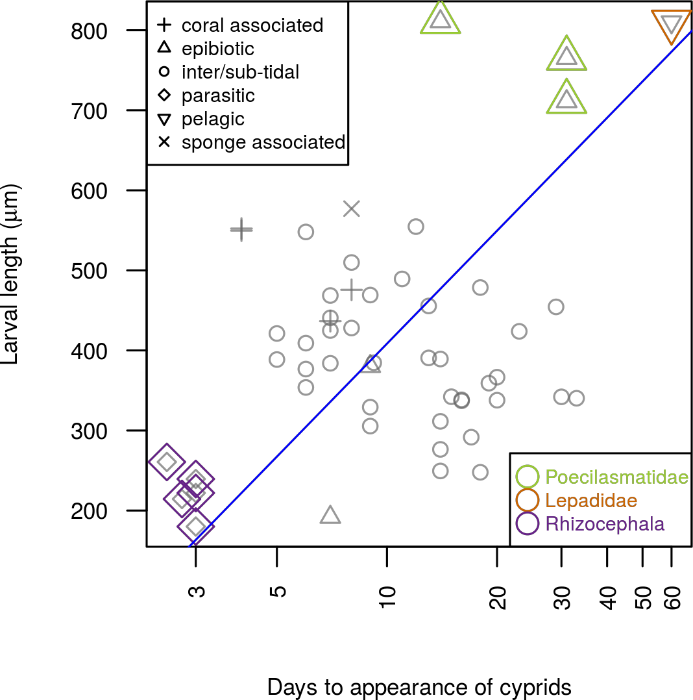


**Fig. G:** **Regression of larval length on nauplius duration.** Nauplius duration based on data of duration for earliest appearance of cypris larvae. Nauplius duration from species reared under 20°C were removed from the regression (detailed data on interspecific nauplius duration can be found in Fig. H). Range major axis regression was used, and the *R*^2^ of the model is 13.38% with a *p*-value of 0.008. Same regression model ran with species with extreme morphology removed returned a non-significant model (*p*-value=0.418). Note the logarithmic scale of x-axis.


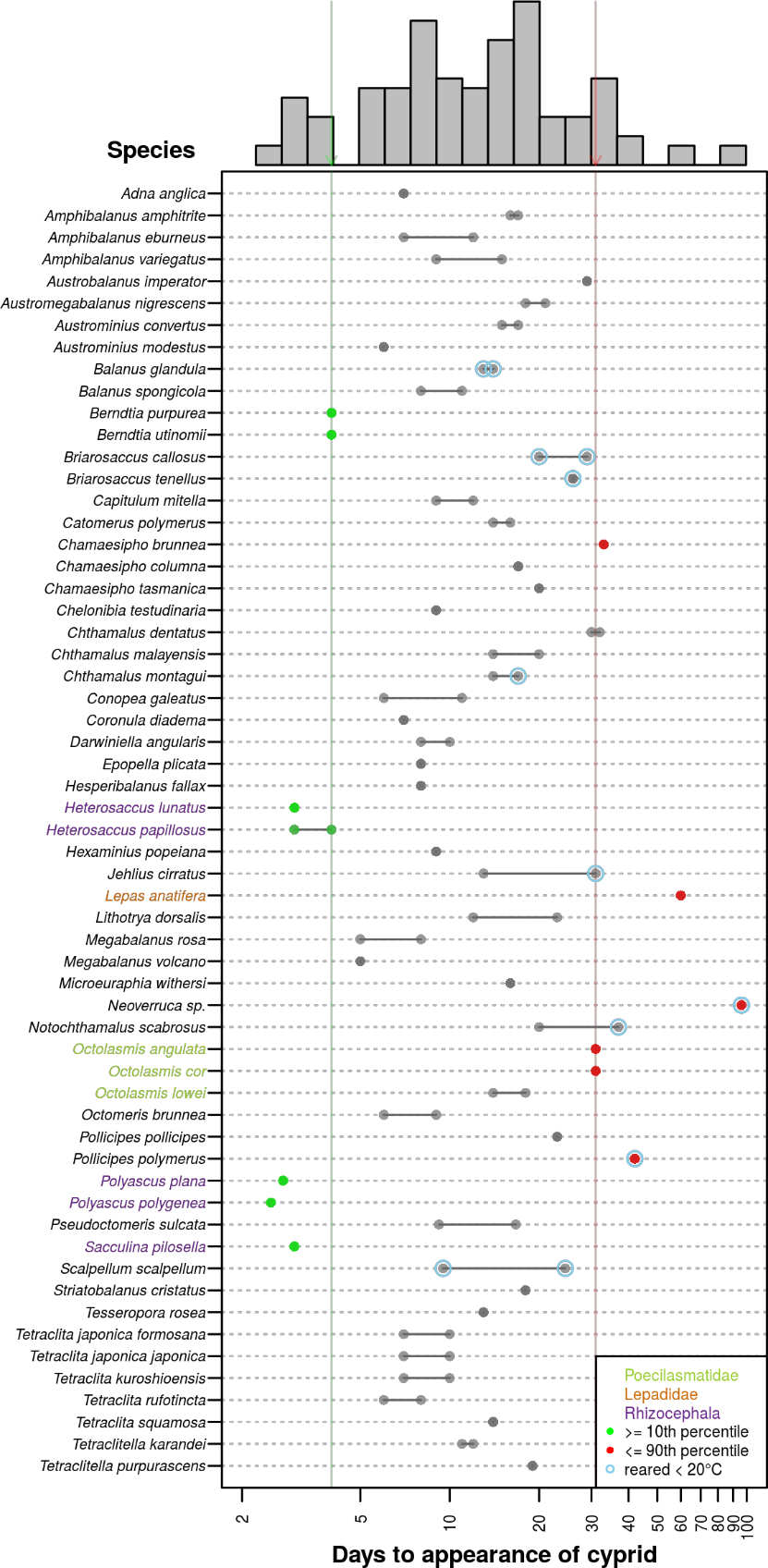


**Fig. H: Interspecific differences in barnacle planktonic larval duration (PLD).** PLD for each species is expressed as single data point representing the cyprid’s first appearance or range with two data points connected by a black line, depending on the availability of data. Most of the species have PLD between 4 (10^th^ percentile) and 31 (90^th^ percentile) days, indicated by green and red lines, respectively.

### Supplementary references

[1] Moyse J. The larval stages of *Acasta spongites* and *Pyrgoma anglicum* (Cirripedia). Proceedings of the Zoological Society of London. 1961;137(3):371–392.

[2] Egan EA, Anderson DT. Larval development of *Balanus amphitrite* Darwin and *Balanus variegatus* Darwin (Cirripedia, Balanidae) from New South Wales, Australia. Crustaceana. 1986;51(2):188–207.

[3] Costlow JD, Bookhout CG. Larval development of *Balanus eburneus* in the laboratory. The Biological Bulletin. 1957;112(3):313–324.

[4] Jones LWG, Crisp DJ. The larval stages of the barnacle *Balanus improvisus* Darwin. Proceedings of the Zoological Society of London. 1954;123(4):765–780.

[5] Lee C, Shim JM, Kim CH. Larval development of *Balanus reticulatus* Utinomi, 1967 (Cirripedia, Thoracica) and a comparison with other barnacle larvae. Journal of Plankton Research. 1999;21(11):2125–2142.

[6] Lang WH. Larval development of shallow water barnacles of the Carolinas (Cirripedia: Thoracica) with keys to the naupliar stages. National Oceanic and Atmospheric Administration; 1979. NMFS Circular 421.

[7] Egan EA, Anderson DT. Larval development of the coronuloid barnacles *Austrobalanus imperator* (Darwin), *Tetraclitella purpurascens* (Wood) and *Tesseropora rosea* (Krauss) (Cirripedia, Tetraclitidae). Journal of Natural History. 1988;22(5):1379–1405.

[8] Egan E, Anderson D. Laval development of the Megabalanine Balanomorph *Austromegabalanus nigrescens* (Lamarck)(Cirripedia, Balanidae). Marine and Freshwater Research. 1987;38(4):511–522.

[9] Egan E, Anderson D. Larval development of *Elminius covertus* Foster and *Hexaminius popeiana* Foster (Cirripedia: Archaeobalanidae: Elminiinae) reared in the laboratory. Marine and Freshwater Research. 1985;36(3):383–404.

[10] Barker MF. Culture and morphology of some New Zealand barnacles (Crustacea: Cirripedia). New Zealand Journal of Marine and Freshwater Research. 1976;10(1):139–158.

[11] Chan BKK, Kolbasov GA, Cheang CC. Cryptic diversity of the acrothoracican barnacle *Armatoglyptes taiwanus* in the Indo-Pacific waters, with description of a new species from the Mozambique Channel collected from the MAINBAZA cruise. Zoosystema. 2012;34(1):5–20.

[12] Barnes H, Costlow JD. The larval stages of *Balanus balanus* (L.) da Costa. Journal of the Marine Biological Association of the United Kingdom. 1961;41(01):59–68.

[13] Branscomb ES, Vedder K. A description of the naupliar stages of the barnacles, *Balanus glandula* Darwin, *Balanus cariosus* Pallas, and *Balanus crenatus* Bruguière (Cirripedia, Thoracica). Crustaceana. 1982;42(1):83–95.

[14] Brown SK, Roughgarden J. Growth, morphology, and laboratory culture of larvae of *Balanus glandula* (Cirripedia: Thoracica). Journal of Crustacean Biology. 1985;5(4):574–590.

[15] Barnes H, Barnes M. the naupliar stages of *Balanus nubilis* Darwin. Canadian Journal of Zoology. 1959;37(1):15–23. [doi:10.1139/z59-003](http://dx.doi.org/10.1139/z59-003).

[16] Korn OM, Elfimov AS, Skreptsova NV. Larval development of a barnacle, *Balanus spongicola* (Cirripedia: Balanidae) reared in the laboratory. Journal of the Marine Biological Association of the UK. 2001;81(05):775–779.

[17] Hawkes C, Meyers T, Shirley T. Larval biology of *Briarosaccus callosus* Boschma (Cirripedia: Rhizocephala). Proceedings of the Biological Society of Washington. 1985;98(4):935–944.

[18] Walossek D, Høeg JT, Shirley TC. Larval development of the rhizocephalan cirripede *Briarosaccus tenellus* (Maxillopoda: Thecostraca) reared in the laboratory: a scanning electron microscopy study. Hydrobiologia. 1996;328(1):9–47.

[19] Egan EA, Anderson DT. Larval development of the chthamaloid barnacles *Catomerus polymerus* Darwin, *Chamaesipho tasmanica* Foster & Anderson and *Chthamalus antennatus* Darwin (Crustacea: Cirripedia). Zoological Journal of the Linnean Society. 1989;95(1):1–28.

[20] Zardus JD, Hadfield MG. Larval development and complemental males in *Chelonibia testudinaria*, a barnacle commensal with sea turtles. Journal of Crustacean Biology. 2004;24(3):409–421.

[21] Kim MH, Yamaguchi T. Larval development and phyogenetic relationship between *Chthamalus challengeri* and *Euraphia pilsbryi* (subclass Cirripedia, suborder Balanomorpha, family Chthamalidae). Marine Fouling. 1996;12(2):1–23.

[22] Achituv Y. The larval development of *Chthamalus dentatus* Krauss (Cirripedia) from South Africa. Crustaceana. 1986;51(3):259–269.

[23] Yan Y, Chan BKK. Larval development of *Chthamalus malayensis* (Cirripedia: Thoracica) reared in the laboratory. Journal of the Marine Biological Association of the United Kingdom. 2001;81(04):623–632.

[24] Burrows MT, Hawkins SJ, Southward AJ. Larval development of the intertidal barnacles *Chthamalus stellatus* and *Chthamalus montagui*. Journal of the Marine Biological Association of the United Kingdom. 1999;79(1):93–101.

[25] Yan Y, Chan BKK. Larval morphology of a recently recognized barnacle, *Chthamalus neglectus* (Cirripedia: Thoracica: Chthamalidae), from Hong Kong. Journal of Crustacean Biology. 2004;24(4):519–528.

[26] Dalley R. The larval stages of the oceanic, pedunculate barnacle *Conchoderma auritum* ( L .) ( Cirripedia : Thoracica ). Crustaceana. 1984;46(1):39–54.

[27] Molenock J, Gomez ED. Larval stages and settlement of the barnacle *Balanus (conopea) galeatus* (L.) (Cirripedia Thoracica). Crustaceana. 1972;23(1):100–108.

[28] Nogata Y, Matsumura K. Larval development and settlement of a whale barnacle. Biology Letters. 2006;2(1):92–3.

[29] Lee C, Kim CH. Larval development of *Balanus albicostatus* Pilsbry (Cirripedia, Thoracica) reared in the laboratory. Journal of Experimental Marine Biology and Ecology. 1991;147(2):231–244.

[30] Karande AA. The nauplii of *Balanus kondakovi*. Proceedings of Indian Academy of Sciences, Section B. 1979;88(1):73–83.

[34] Sandison EE, Day JH. The identification of the nauplii of some South African barnacles with notes on their life histories. Transactions of the Royal Society of South Africa. 1954;34(1):69–101.

[32] Korn OM, Elfimov AS. Larval development of a warm-water immigrant barnacle, *Solidobalanus fallax* (Cirripedia: Archaeobalanidae) reared in the laboratory. Journal of the Marine Biological Association of the United Kingdom. 1999 12;79(6):1039–1044.

[33] Barnes H, Barnes M. The naupliar stages of *Balanus hesperius* Pilsbry. Canadian Journal of Zoology. 1959;37(3):237–244.

[34] Walker G, Lester RJG. Effect of salinity on development of larvae of *Heterosaccus lunatus* (Cirripedia: Rhizocephala). Journal of Crustacean Biology. 1998;18(4):650–655.

[35] Ponomarenko E, Korn O, Rybakov A. Larval development of the parasitic barnacle *Heterosaccus papillosus* ( Cirripedia : Rhizocephala : Sacculinidae ) studied under laboratory conditions. Journal of the Marine Biological Association of the United Kingdom. 2005;85:921–928.

[36] Venegas RM, Ortíz V, Olguín A, Navarrete SA. Larval development of the intertidal barnacles *Jehlius cirratus* and *Notochthamalus scabrosus* (Cirripedia: Chthamalidae) under laboratory conditions. Journal of Crustacean Biology. 2000;20(3):495–504.

[37] Moyse J. Larvae of lepadomorph barnacles. In: Southward AJ, editor. Barnacle Biology. No. 5 in Crustacean Issues. Netherlands: A. A. Balkema; 1987. p. 329–362.

[38] Tunnicliffe V, Southward AJ. Growth and breeding of a primitive stalked barnacle *Leucolepas longa* (Cirripedia : Scalpellomorpha : Eolepadidae : Neolepadinae) inhabiting a volcanic seamount off Papua New Guinea. Journal of the Marine Biological Association of the United Kingdom. 2004;84(1):121–132.

[39] Dineen JF Jr. The larval stages of *Lithotrya dorsalis* (Ellis & Solander, 1786): a burrowing Thoracican barnacle. The Biological Bulletin. 1987;172(3):284–298.

[40] Miller KM, Roughgarden J. Descriptions of the larvae of *Tetraclita rubescens* and *Megabalanus californicus* with a comparison of the common barnacle larvae of the Central California coast. Journal of Crustacean Biology. 1994;14(3):579–600.

[41] Kado R, Hirano R. Larval development of two Japanese Megabalanine barnacles, *Megabalanus volcano* (Pilsbry) and *Megabalanus rosa* (Pilsbry) (Cirripedia, Balanidae), reared in the laboratory. Journal of Experimental Marine Biology and Ecology. 1994;175(1):17–41.

[42] Lee C, Shim JM. Larval development of *Membranobalanus koreanus* Kim & Kim, 1983, reared in the laboratory (Cirripedia, Thoracica). Crustaceana. 2000;73(4):393–406.

[43] Sandison EE. The naupliar stages of *Balanus pallidus stutsburi* Darwin and *Chthamalus aestuarii* Stubbings (Cirripedia Thoracica). Crustaceana. 1967;13(2):161–174.

[44] Li H, Miao S, Yan Y, Yu X, Zhang L. Larval development of the barnacle, *Microeuraphia withersi* (Cirripedia, Thoracica, Chthamalidae) reared in the laboratory. Crustaceana. 2011;84(2):129–152.

[45] Stewart BA, Cook PA, Achituv Y. Naupliar stages of the coral inhabiting barnacle *Savignium milleporum* (Darwin)(Cirripedia: Pyrgomatidae) from the Gulf of Eilat, Red Sea. Bulletin of Marine Science. 1989;45(1):164–173.

[46] Watanabe H, Kado RO, Tsuchida SP, Miyake HP, Kyo MP, Kojima S. Larval development and intermoult period of the hydrothermal vent barnacle *Neoverruca* sp. Journal of the Marine Biological Association of the United Kingdom. 2004;84(4):743–745.

[47] Yap FC, Wong WL, Maule AG, Brennan GP, Lim LHS. Larval development of the pedunculate barnacles *Octolasmis angulata* Aurivillius 1894 and *Octolasmis cor* Aurivillius 1892 (Cirripedia: Thoracica: Poecilasmatidae) from the gills of the mud crab, *Scylla tranquebarica* Fabricius, 1798. Arthropod Structure and Development. 2014;44(3):253–279.

[48] Lang WH. The larval development and metamorphosis of the pedunculate barnacle *Octolasmis mülleri* (Coker, 1902) reared in the laboratory. Biological Bulletin. 1976;150(2):255–267.

[49] Kim MH, Yamaguchi T. Larval development of *Octomeris brunnea* Darwin (Cirripedia, Chthamalidae, Euraphiinae) reared in the laboratory. Sessile Organisms. 1998;14(2):1–17.

[50] Molares J, Tilves F, Pascual C. Larval development of the pedunculate barnacle *Pollicipes cornucopia* (Cirripedia: Scalpellomorpha) reared in the laboratory. Marine Biology. 1994;120(2):261–264.

[51] Lewis CA. Development of the gooseneck barnacle *Pollicipes polymerus* (Cirripedia: Lepadomorpha): fertilization through settlement. Marine Biology. 1975;32(2):141–153.

[52] Tu TH, Chan BKK, Jeng MS. Larval development and sex ratio variation of *Polyascus plana* (Cirripedia: Rhizocephala), a parasite of the crab *Grapsus albolineatus*, in Taiwan. Bulletin of Marine Science. 2009;84(3):331–349.

[53] Korn OM, Rybakov AV, Kashenko SD. Larval development of the rhizocephalan *Sacculina polygenea* (Crustacea: Cirripedia). Russian Journal of Marine Biology. 2000;26(5):373–377.

[54] Kado R, Kim MH. Larval development of *Octomeris sulcata* Nilsson-Cantell (Cirripedia: Thoracica: Chthamalidae) from Japan and Korea. Hydrobiologia. 1996;325(1):65–76.

[55] Collis SA, Walker G. The morphology of the naupliar stages of *Sacculina carcini* (Crustacea: Cirripedia: Rhizocephala). Acta Zoologica. 1994;75(4):297–303.

[56] Korn OM, Rybakov AV. Larval development in the rhizocephalan barnacle *Sacculina pilosella*. Russian Journal of Marine Biology. 2001;27(3):177–179.

[57] Kaufmann R. Zur embryonal-und larvalentwicklung von *Scalpellum scalpellum* L.(crust. cirr.). Zeitschrift für Morphologie und Ökologie der Tiere. 1965;55(2):161–232.

[58] Pyefinch KA. Methods of identification of the larvae of *Balanus balanoides* (L.), *B. crenatus* Brug. and *Verruca stroemia* O. F. Müller. Journal of the Marine Biological Association of the United Kingdom. 1948;27(2):451–463.

[59] Lee C, Shim JM, Jeong MK, Kim CH. Larval development of *Chirona cristatus* (Cirripedia: Thoracica) reared in the laboratory. Animal Systematics, Evolution and Diversity. 2002;18(1):35–48.

[60] Crisp DJ. A comparison between the reproduction of high- and low-latitude barnacles, including *Balanus balanoides* and *Tetraclita (Tesseropora) pacifica*. In: Thompson MF, Sarojini R, Nagabhushanam R, editors. Biology of benthic marine organisms: techniques and methods as applied to the Indian Ocean. New Delhi: Oxford and IBH Publishing Co; 1986. p. 69–84.

[61] Barnes M, Achituv Y. The nauplius stages of the cirripede *Tetraclita squamosa rufotincta* Pilsbry. Journal of Experimental Marine Biology and Ecology. 1981;54(2):149 – 165.

[62] Chan BKK. Studies on *Tetraclita squamosa* and *Tetraclita japonica* (Cirripedia: Thoracica) II: larval morphology and development. Journal of Crustacean Biology. 2003;23(3):522–547.

[63] Karande AA. Larval development of the barnacle *Tetraclitella karandei* reared in the laboratory. Biological Bulletin. 1974;146(2):249–257.

[64] Pérez-Losada M, Harp M, Høeg JT, Achituv Y, Jones D, Watanabe H, et al. The tempo and mode of barnacle evolution. Mol Phylogenet Evol. 2008;46(1):328 – 346.

[65] Revell LJ. phytools: an R package for phylogenetic comparative biology (and other things). Methods in Ecology and Evolution. 2012;3(2):217–223.
